# Effect of Cigarette Constituent Messages With Engagement Text on Intention to Quit Smoking Among Adults Who Smoke Cigarettes: A Randomized Clinical Trial

**DOI:** 10.1001/jamanetworkopen.2021.0045

**Published:** 2021-02-24

**Authors:** Adam O. Goldstein, Kristen L. Jarman, Sarah D. Kowitt, Tara L. Queen, Kyung Su Kim, Bonnie E. Shook-Sa, Paschal Sheeran, Seth M. Noar, Leah M. Ranney

**Affiliations:** 1 Department of Family Medicine, University of North Carolina at Chapel Hill; 2 Lineberger Comprehensive Cancer Center, University of North Carolina at Chapel Hill; 3 Gillings School of Global Public Health, Department of Biostatistics, University of North Carolina at Chapel Hill; 4 Department of Psychology and Neuroscience, University of North Carolina at Chapel Hill; 5 Hussman School of Journalism and Media, Chapel Hill, North Carolina

## Abstract

**Importance:**

The US Food and Drug Administration (FDA) is required to communicate the risks of tobacco constituents to the public. Few studies have addressed how FDA media campaigns can effectively communicate about cigarette smoke constituents.

**Objective:**

To examine whether messages about cigarette smoke constituents are effective in reducing smoking intentions and behaviors among adults who smoke.

**Design, Setting, and Participants:**

This randomized clinical trial enrolled participants who were aged between 18 and 65 years, were English speakers, were living in the United States, and who smoked at least 100 cigarettes during their lifetime and now smoked every day or some days. Participants received daily messages via email for 15 days. Participants were randomized to 1 of 2 message conditions or a control group and reported their previous-day smoking behaviors daily. Follow-up surveys were conducted on days 16 and 32. Data were collected from June 2017 to April 2018 and analyzed from April to September 2018.

**Interventions:**

The 3 groups were (1) constituent plus engagement messages (eg, “Cigarette smoke contains arsenic. This causes heart damage.”) that included the FDA as the source and engagement text (eg, “Within 3 months of quitting, your heart and lungs work better. Ready to be tobacco free? You can quit. For free nicotine replacement, call 1-800-QUIT-NOW”); (2) constituent-only messages that did not list the FDA as the source or include engagement text; and (3) a control condition with messages about littering cigarette butts.

**Main Outcomes and Measures:**

The primary outcome was the change in quit intentions (range, 1-4, with higher scores indicating stronger intentions) from pretest to day 16. Secondary outcome measures included daily smoking behaviors and quit attempts.

**Results:**

A total of 789 participants (mean [SD] age, 43.4 [12.9] years; 483 [61.2%] women; 578 [73.3%] White; 717 [90.9%] non-Hispanic) were included in the study. The mean (SD) quit intention score was 2.5 (0.9) at pretest. Mean (SE) change in quit intention score from pretest to day 16 was 0.19 (0.07) points higher in the constituent plus engagement condition than in the control condition (*P* = .005) and 0.23 (0.07) points higher in the constituent-only condition compared with the control condition (*P* = .001). Participant reports of cigarettes smoked, forgone, and butted out were similar across study conditions at baseline and did not differ significantly at days 16 and 32 across study conditions. Viewing more messages was associated with an estimated decrease of 0.15 (SE, 0.01) cigarettes smoked per day per message viewed overall across conditions.

**Conclusions and Relevance:**

To our knowledge, this is the first longitudinal test of cigarette constituent campaign messages in a national sample of adults who currently smoke. Messages about cigarette smoke constituents, with or without engagement text and source information, increased participants’ intentions to quit, lending support to FDA efforts to educate consumers about such constituents.

**Trial Registration:**

ClinicalTrials.gov Identifier: NCT03339206

## Key Points

**Question:**

Do cigarette constituent messages with US Food and Drug Administration sourcing and engagement text (ie, encouragement to quit) increase intentions to quit more than cigarette constituent messages alone or control messages?

**Findings:**

In this randomized clinical trial of 789 adults who smoke cigarettes, participants assigned to the constituent plus engagement and constituent-only message conditions demonstrated increased quit intentions from preintervention to postintervention relative to participants in the control condition.

**Meaning:**

These findings indicate that messages about cigarette smoke constituents increased smokers’ intentions to quit, which can inform national efforts to communicate harmful constituents in cigarette smoke among adults who smoke.

## Teaser

This randomized clinical trial examines whether messages about cigarette smoke constituents are effective in reducing smoking intentions and behaviors among adults who smoke.

## Introduction

Cigarette smoking continues to cause hundreds of thousands of deaths annually in the United States.^1^ While smoking rates have reached the lowest level ever recorded among US adults, 34.2 million adults still smoke cigarettes.^2^ Smoking is higher among socioeconomically disadvantaged populations, and these populations experience disproportionately higher smoking-related health effects.^3^ Smoking results in a large annual loss in US economic productivity ($170 billion) and higher medical care expenditures ($133 billion), accentuating the need for stronger public health solutions.^4^

Population-based tobacco control interventions are associated with a reduction in tobacco use among both adults and youth and increased rates of quitting smoking.^5^ Tobacco control strategies include increasing tobacco prices, implementing smoke-free laws, improving access to evidence-based cessation treatment, and deploying hard-hitting media campaigns.^4^ Research shows that mass media campaigns have a wide population reach and can change smoking behaviors and be cost-effective.^6,7,8^ The duration, intensity, message design, and targeting of the media campaign to a specific population play an important role in the success and effectiveness of the campaign.^7^

Since 2009, the US Food and Drug Administration (FDA) has taken significant steps to protect the public from the dangers of tobacco through new regulations.^9^ One FDA requirement is to communicate about harmful and potentially harmful constituents found in tobacco products and tobacco smoke.^10^ Currently, the FDA is pursuing methods to ensure that the public understands the real and potential risks of tobacco use by including messages about tobacco constituents in public health campaigns. Research examining cigarette smoke constituent messages suggests that there is a substantial misunderstanding of the source of harmful constituents, and awareness that certain chemicals are contained in tobacco smoke is low.^11,12^ Another study found that the constituents that could most effectively discourage cigarette smoking have familiar names, like arsenic and formaldehyde.^13^ In a longitudinal study of adults in the US, awareness of chemicals in cigarette smoke did not increase for either those who smoke or those who do not smoke in surveys conducted in 2014 and 2017.^11^ Furthermore, the correct belief that harmful chemicals in cigarette smoke come from burning the cigarette decreased over this period, meaning that adults’ understanding of the source of the risk from tobacco smoking may have declined over time.^11^

A small number of studies have incorporated cigarette smoke constituent messages into antismoking advertisements.^14,15,16^ Research shows that incorporating some message elements, such as graphic images, and using familiar constituents, such as arsenic, may be particularly effective.^16^ However, only 1 randomized clinical trial (RCT) has examined the impact of cigarette smoke constituent messaging that appears on packs on smoking outcomes, and to our knowledge, no RCT has examined the impact of a cigarette smoke constituent communications campaign on smoking outcomes.^17^

The purpose of this study was to conduct an RCT on constituent message elements (ie, image, FDA source, and engaging text about quitting) to determine impact on quit intentions. Prior research has shown the importance of source credibility (ie, presenting the FDA logo) on attitudes and behavioral intentions.^14,15^ Engagement text about quitting, including information on the benefits of quitting^18,19,20,21^ with an interrogative cue,^22,23^ a self-efficacy cue,^18,19^ and quitline information^24,25^ can all enhance the impact of messages.^19^ We hypothesized that constituent messages with FDA source and engagement text (ie, encouragement to quit) would increase intentions to quit more than constituent messages alone or control messages.

## Methods

We preregistered our study at ClinicalTrials.gov. The University of North Carolina institutional review board approved all study procedures, and all participants provided informed consent online prior to participation. This report follows the Consolidated Standards of Reporting Trials (CONSORT) reporting guideline for randomized studies. We also prespecified our analysis plan according to guidelines in Gamble et al.^26^ The trial protocol is available in Supplement 1.

### Participants

We conducted a parallel, 3-condition RCT with a national sample of US adults who smoke cigarettes. Participants were aged between 18 and 65 years, spoke English, and currently smoked (defined as having smoked ≥100 cigarettes during their lifetime and now smoking every day or some days). We excluded people who were currently enrolled in smoking cessation programs, people who were currently using pharmacotherapy for smoking cessation, and people who had participated in a smoking study in the previous 3 months. Given that our trial was delivered virtually, we also excluded people who did not have access to the internet at home or work, people who were not able to complete a survey on a computer, and people who did not think they would be able to regularly complete surveys delivered via email. We recruited participants to take a screening survey for the study from June 2017 to April 2018 using 2 methods: (1) a previous, nationally representative survey on tobacco use^27^ and (2) targeted social media advertisements.

### Procedures

**Figure 1.**
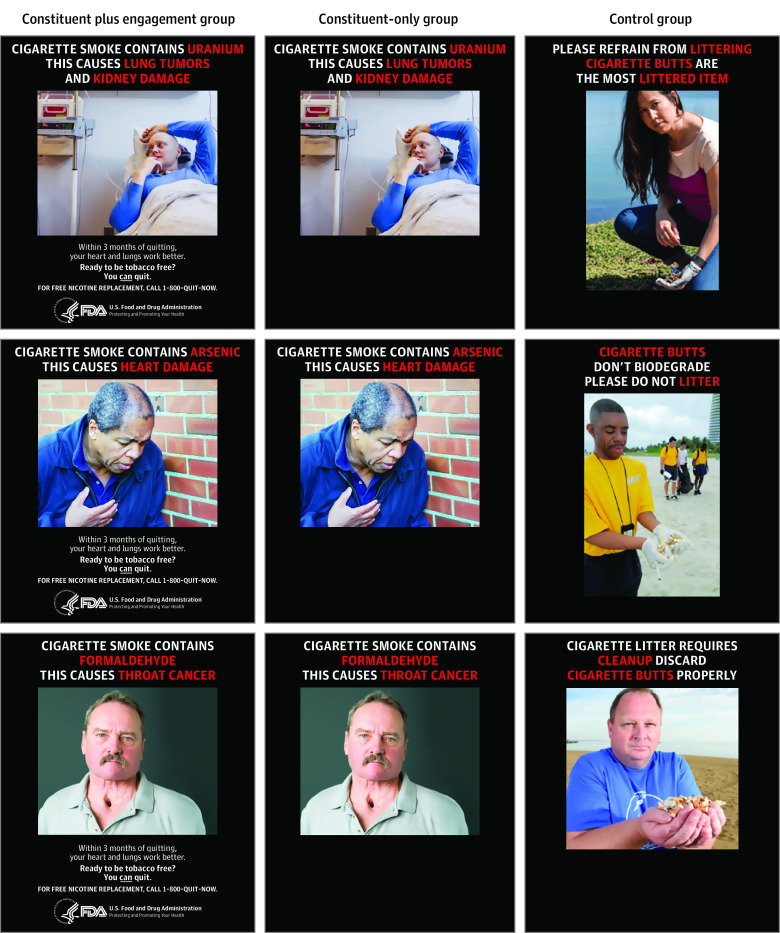
Message by Condition Images paired with constituent messages are stock images that were purchased by the researchers; images paired with littering messages are (from top to bottom) from the *Alameda Magazine*,^28^ Getty Images, and the *Herald Sun*.^29^ These images were either publicly available or purchased by the researchers.

We referred all potential participants to an online screener, through which we assessed their eligibility. We then contacted eligible participants by email and invited them to take part in our study by completing the pretest survey (day 0). At the end of the pretest questionnaire, we assigned participants to 1 of 3 conditions, using the randomization function from Qualtrics software (Qualtrics). We set the randomization to ensure that each participant had an equal likelihood of being assigned to each condition, so that each condition would have a similar number of participants. In our 2 experimental conditions, we presented messages about 5 tobacco constituents (ie, lead, uranium, arsenic, formaldehyde, and ammonia). An example message read: “Cigarette smoke contains ammonia. This causes breathing problems.” The first condition included tobacco constituent messages with the FDA logo and quit information that read: “Within 3 months of quitting, your heart and lungs work better. Ready to be tobacco free? You can quit. For free nicotine replacement, call 1-800-QUIT-NOW.” We labeled this condition the constituent plus engagement condition. The second condition included tobacco constituent messages without the FDA logo or any quit information, which we labeled the constituent-only condition. The third condition functioned as our control and included messages about littering cigarette butts (Figure 1; eFigure 1 in Supplement 2).^28,29^

We chose these 5 tobacco constituents because they performed well in previous online studies.^30,31,32^ Messages in these conditions also featured an image of an individual displaying the health effects of the tobacco constituent. For example, in the message about ammonia (which causes breathing problems), we presented a man receiving oxygen through his nose. We used the same images for the constituent plus engagement and constituent-only conditions because we only wanted to manipulate the presence of a source and quit information.

In our control condition, we presented 5 different messages about littering (eg, “Cigarette butts don’t biodegrade. Please do not litter”). We matched images in this condition to the constituent plus engagement and constituent-only conditions by gender and race/ethnicity of the featured individual in the image (Figure 1). For instance, if an image in the constituent plus engagement and constituent-only conditions featured a Black man looking at the camera, then the image in the control condition also featured a Black man looking at the camera.

Participants were not informed about the possible interventions to which they may have been assigned. Researchers were not masked to the condition that participants had been assigned to; however, all outcome measures were assessed via online survey.

On days 1 to 15, we delivered 1 message per day to participants by email. We required participants to view the message—which contained text and an image—for 10 seconds and then asked questions about smoking and littering behaviors, message-elicited affect, and perceived message credibility and effectiveness. Each condition included a total of 5 messages, and we repeated the same messages 3 times within conditions. We used block randomization to control for the order of each stimulus.

On day 16 and day 32, participants answered posttest survey items. For their participation, participants received up to $150, depending on the number of surveys completed. We used SurveySignal^33^ to automatically email participants the daily and posttest surveys and Qualtrics software (Qualtrics) for programming and collecting data from the surveys.

### Internal and External Pilots

Before conducting the full RCT, we conducted 2 pilot tests to ensure the survey flow and procedures worked correctly. First, we internally piloted the study with 19 members from our research team. Second, we conducted an external pilot by inviting 40 eligible participants, 19 of whom enrolled in the study. We identified a diverse set of participants based on gender, race/ethnicity, time zone, and recruitment pool (ie, telephone survey on tobacco use and social media advertisements). No major issues were reported. There were no changes to the trial design once data collection started.^34^

### Outcome Measures

We used validated items for all surveys (pretest survey, daily surveys, 2 posttest surveys). The pretest and posttest surveys assessed demographic characteristics as well as most primary and secondary outcomes. The first posttest survey also included quality assurance measures to make sure participants saw all messages and had no problems responding to questions.

The primary a priori trial outcome was change in quit intentions from the pretest to the first posttest survey (day 16 of the study). We asked participants 3 questions: (1) “How interested are you in quitting smoking in the next month?”, (2) “How much do you plan to quit smoking in the next month?”, and (3) “How likely are you to quit smoking in the next month?” Response options ranged from very interested (coded as 4) to not at all interested (coded as 1). We averaged responses to create a mean score, where higher scores indicated higher intentions, and calculated the difference between the posttest and pretest measures.^35^

We assessed several smoking-related behaviors as daily secondary outcomes, including number of cigarettes smoked each day (”Yesterday, from the time you woke up until noon, how many cigarettes did you smoke?” and “Yesterday, from noon until you went to sleep, how many cigarettes did you smoke?”), number of cigarettes forgone each day (“How many times yesterday did you stop yourself from having a cigarette because you wanted to smoke less?”), and number of cigarettes butted out each day (“How many times yesterday did you butt out a cigarette before you finished because you wanted to smoke less?”). We also assessed participants’ reported quit attempts at pretest, posttest 1 (day 16), and posttest 2 (day 32). In the pretest survey, we asked participants, “How many times during the past 12 months have you stopped smoking for 1 day or longer because you were trying to quit smoking?”, and in the posttest surveys, we asked participants, “Since you started this study, how many times have you stopped smoking for 1 day or longer because you were trying to quit smoking?” There were no changes to methods or trial outcomes once the study commenced.

### Statistical Analysis

#### Primary Analysis

For our primary outcome, we used general linear modeling to examine study arm differences in changes in quit intentions after controlling for the number of messages viewed. Because this model included a variable measured postrandomization, we also conducted the intention-to-treat (ITT) analysis as a sensitivity analysis without controlling for the number of messages viewed (eTable 1 in Supplement 2); results using both approaches were similar. We used multiple imputation to account for missing data (eAppendix in Supplement 2). All participants reported a quit intention at pretest, and 699 (92.9%) reported quit intentions at posttest 1. Number of messages viewed, age, gender, income status, education, and nicotine dependence scores all informed the multiple imputation of difference in quit intentions. All analyses used SAS version 9.4 (SAS Institute). We set critical α = .05 and used 2-tailed statistical tests. Data were analyzed from April to September 2018.

#### Secondary Analyses

For secondary outcome analyses, we used multilevel modeling to account for the nested structure of these data and to examine study arm differences in these daily behaviors after controlling for the number of messages viewed as of that day. As with the primary outcome, we also conducted ITT analyses as sensitivity analyses without controlling for the number of messages viewed (eTable 2 and eTable 3 in Supplement 2). As in our primary outcome, results using both approaches were similar. Multilevel modeling is well equipped to handle a moderate amount of missing data because this modeling does not assume an equal number of observations. As a result, all cases were used for secondary outcomes analysis, which aligns with our ITT approach.^36^

## Results

### Participant Characteristics

**Figure 2.**
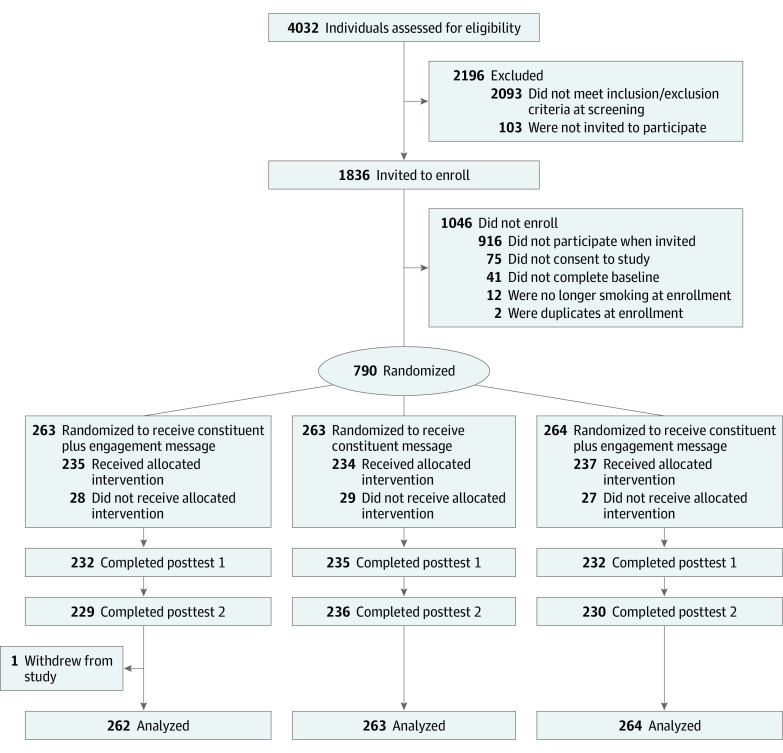
Study Flow Diagram Not all eligible participants were invited because enrollment goals were met before all eligible potential participants were invited. To receive the allocated intervention, participants had to complete at least 1 of the daily questionnaires.

The final analytic sample size for the study was 789 participants (mean [SD] age, 43.4 [12.9] years; 483 [61.2%] women; 578 [73.3%] White; 717 [90.9%] non-Hispanic) (Table 1). Race and ethnicity classifications were defined by the participants. All participants were enrolled in the study between January and June 2018. Trial recruitment ended once we reached our enrollment goal. The flowchart of participation is presented in Figure 2. Participants resided in 46 US states and the District of Columbia. Most participants had completed some college (275 [34.9%]), a Bachelor’s degree (160 [20.3%]), or had a high school diploma or equivalent (170 [21.6%]). A substantial portion of participants (123 [15.6%]) reported incomes below the 2017 federal poverty line based on their household size.^37^ Most of the sample identified as straight or heterosexual (691 [87.6%]), while 98 (12.4%) identified as gay, lesbian, bisexual, or other. At baseline, 695 participants (88.1%) reported smoking every day, while 94 (11.9%) reported smoking some days. The mean (SD) Fagerstrom Nicotine Dependence (FTND) Score was 5.2 (2.4) of 10, indicating moderate nicotine dependence on average.^38^ Quit intention scores ranged from 1 to 4, and the mean (SD) score was 2.5 (0.9), indicating mild interest in quitting. The mean (SD) numbers of cigarettes smoked, butted out, and forgone in the previous day were 16 (10.1), 1.3 (2.5), and 2.0 (3.2), respectively. Finally, the mean (SD) number of self-reported quit attempts at pretest was 3.3 (16.5).

**Table 1.** Participant Characteristics at Pretest

Characteristic	No. (%)			
	Condition			Total sample (N = 789)
	Constituent and engagement (n = 262)	Constituent only (n = 263)	Control (n = 264)	
Gender				
Male	99 (37.8)	96 (36.5)	106 (40.2)	301 (38.2)
Female	161 (61.5)	166 (63.1)	156 (59.1)	483 (61.2)
Nonconforming	2 (0.8)	1 (0.4)	2 (0.8)	5 (0.6)
Age, mean (SD), y	43.9 (12.8)	42.8 (12.7)	43.3 (13.1)	43.4 (12.9)
Race				
White	192 (73.3)	181 (68.8)	205 (77.7)	578 (73.3)
Black or African American	52 (19.9)	63 (24.0)	49 (18.6)	164 (20.8)
Other race^a^	18 (6.9)	19 (7.2)	10 (4.8)	47 (6.0)
Ethnicity				
Latino/Hispanic	22 (8.4)	27 (10.3)	23 (8.7)	72 (9.1)
Non-Latino/Hispanic	240 (91.6)	236 (89.7)	241 (91.29)	717 (90.9)
Education				
<High school	16 (6.1)	14 (5.32)	8 (3.0)	38 (4.8)
G12, GED, or high school diploma	63 (24.1)	51 (19.4)	56 (21.2)	170 (21.6)
Some college	86 (32.8)	89 (33.84)	100 (37.9)	275 (34.9)
Associate degree	32 (12.2)	35 (13.31)	30 (11.4)	97 (12.3)
Bachelor’s degree	46 (17.6)	57 (21.7)	57 (21.6)	160 (20.3)
Graduate or professional degree	19 (7.3)	17 (6.5)	13 (4.9)	49 (6.2)
Income status				
<Poverty line	32 (12.2)	41 (15.6)	50 (18.9)	123 (15.6)
>Poverty line	229 (87.4)	222 (84.4)	214 (81.1)	665 (84.3)
Missing	1 (0.4)	0	0	1 (0.1)
Sexual orientation				
Straight or heterosexual	229 (87.4)	230 (87.5)	232 (87.9)	691 (87.6)
Gay, lesbian, or bisexual	32 (12.2)	32 (12.2)	30 (11.4)	94 (11.9)
Other	1 (0.4)	1 (0.4)	2 (0.9)	4 (0.5)
Current cigarette smoking				
Some days	26 (9.9)	34 (12.9)	34 (12.9)	94 (11.9)
Everyday	236 (90.1)	229 (87.1)	230 (87.1)	695 (88.1)
Fagerstrom nicotine dependence score, mean (SD)^b^	5.1 (2.5)	5.1 (2.3)	5.4 (2.5)	5.2 (2.4)
Quit intentions, mean (SD)^c^	2.5 (0.9)	2.4 (0.9)	2.6 (0.9)	2.5 (0.9)
Cigarettes, mean (SD), No.				
Smoked	16.7 (11.0)	15.4 (10.2)	15.9 (9.1)	16.0 (10.1)
Forgone	1.4 (3.3)	1.1 (1.7)	1.4 (2.2)	1.3 (2.5)
Butted out	2.0 (3.5)	1.9 (2.6)	2.1 (3.3)	2.0 (3.2)
Quit attempts, mean (SD), No.	4.3 (24.7)	2.7 (11.4)	2.8 (9.0)	3.3 (16.5)

Abbreviations: G12, grade 12; GED, general educational development.

^a^ The other race category included participants who identified as American Indian or Alaska Native, Asian, Pacific Islander, or other.

^b^ The response scale for nicotine dependence scores ranged from 0 to 10, with higher scores indicating higher dependence.

^c^ The response scale for quit intentions ranged from 1 to 4, with higher scores indicating higher intentions.

### Messages Viewed

At day 16, participants in the constituent plus engagement condition had viewed a mean (SD) of 10.7 (5.0) messages (71% of the 15 total messages), and participants in the constituent-only condition had viewed a mean (SD) of 10.9 (4.9) messages (73%). Participants in the control condition had viewed a mean (SD) of 10.5 (4.9) messages (70%).

### Primary Outcome

Participants in the constituent plus engagement and constituent-only message conditions reported changes in quit intention scores at posttest 1 (day 16) relative to pretest that were a mean (SD) 0.20 (0.74) points and 0.25 (0.79) points, respectively, higher than participants in the control condition. In our main analysis, changes in quit intentions were a mean (SD) 0.19 (0.07) points higher for the constituent plus engagement condition compared with the control condition (*P* = .005) and a mean (SD) 0.23 (0.07) points higher for the constituent-only condition compared with the control (*P* = .001) (Table 2). There were no significant differences in quit intentions between the constituent plus engagement and constituent-only conditions (eFigure 2 and eTable 4 in Supplement 2).

**Table 2.** Change in Quit Intentions From Pretest to Day 16 and Day 32

Group	Quit intentions			
	Day 16		Day 32	
	Estimate (SE)	*P* value	Estimate (SE)	*P* value
Intercept	−0.10 (0.11)	.39	−0.07 (0.12)	.59
Messages viewed, No.	0.009 (0.009)	.30	0.02 (0.009)	.04
Study condition				
Control	[Reference]	NA	[Reference]	NA
Constituent plus engagement	0.19 (0.07)	.005	0.07 (0.08)	.36
Constituent-only	0.23 (0.07)	.001	0.15 (0.08)	.07

Abbreviation: NA, not applicable.

### Secondary Outcomes

At day 32, there was no significant difference between the change in quit intentions in either study condition compared with the control, but the number of messages viewed was significantly associated with change in quit intention. Each message viewed was associated with a 0.02-point (SE, 0.009; *P* = .04) increase in quit intentions. The number of messages viewed was also significantly associated with the number of cigarettes smoked across the daily surveys. Participants reported smoking 0.15 (SE, 0.01) fewer cigarettes for each 1-unit increase in the number of messages viewed (Table 3). No significant differences were observed between treatment groups for other secondary outcomes, ie, cigarettes smoked, forgone, or butted out (days 0-32) (Table 3). We also found no effect of study condition on quit attempts during the study period (days 0-32) (eTable 5 in Supplement 2).

**Table 3.** Secondary Behavioral Outcomes From Day 0 to 32

Group	Cigarettes					
	Smoked		Forgone		Butted out	
	Estimate (SE)	*P* value	Estimate (SE)	*P* value	Estimate (SE)	*P* value
Intercept	14.85 (0.60)	<.001	0.04 (0.20)	.84	0.22 (0.18)	.21
Messages viewed, No.	−0.15 (0.01)	<.001	0.02 (0.01)	.07	−0.01 (0.01)	.16
Study condition						
Control	[Reference]	NA	[Reference]	NA	[Reference]	NA
Constituent plus engagement	0.19 (0.85)	.83	−0.16 (0.27)	.55	−0.12 (0.25)	.64
Constituent-only	−0.88 (0.85)	.30	0.14 (0.27)	.62	−0.10 (0.25)	.68

Abbreviation: NA, not applicable.

## Discussion

In our theory-driven study, we found that messages that presented information and images on harmful constituents increased smokers’ intentions to quit (our primary outcome) compared with control messages about littering. We also detected no significant differences between our 2 experimental conditions—constituent plus engagement messages (containing a constituent message, an image, FDA source, and engaging text about quitting) and constituent-only messages (including a constituent message and image only). Therefore, our findings suggest that messages featuring a constituent and image are powerful enough to increase smokers’ quit intentions while messages are being received.

Evidence suggests that images, when combined with text, increase message receptivity, enhance subsequent learning, and increase both perceived and actual message effectiveness.^39,40^ This is evident from the successful national antismoking media campaign from the US Centers for Disease Control and Prevention (CDC) *Tips From Former Smokers*. This campaign features graphic images of individuals experiencing long-term health consequences of smoking, and overwhelming evidence supports its acceptability and reach.^41^ Additionally, research shows that constituent messages increase knowledge of constituents, reinforce the harmful health effects of smoking, and discourage people from wanting to smoke cigarettes.^13,31,32,42^ Moreover, these effects may be even more pronounced when constituents are familiar to the public, as in this study. Extending previous research, our study deployed an RCT design that determined that repeated exposure to constituent messages with images increased short-term intentions to quit among adult smokers.^13^

The FDA has made communicating constituent information to the public a priority, consistent with legal mandates and a belief that this can further decrease tobacco consumption.^43^ To date, however, information on the actual impact of this priority have been extremely limited, and no research has examined whether constituent information aids in cessation behaviors. Brewer et al^13^ published studies that showed most US adults did not know much about tobacco constituents and that this knowledge changed little over time.^11^ A follow-up survey showed that researchers could increase constituent knowledge, but no research on the impact of such knowledge on smoking outcomes was conducted.^44,45^ The impact of our intervention on quit intentions was overall modest, but as the first RCT of which we are aware that examines the impact of a public constituent campaign, it provides evidence that communicating constituent information to individuals who use tobacco may help to motivate smoking cessation. It is also reassuring that quit intentions were higher and cigarettes smoked decreased as the number of messages view increased, regardless of condition. Even a small impact could be meaningful on a population level, particularly if policy makers use it as a guide to investigate more effective strategies, channels, and methods to help those wanting to quit. The finding that increased intentions dissipated after the short campaign ended suggests the need to test more sustained and higher-intensity interventions.

While we selected message elements for our constituent plus engagement condition (ie, FDA logo, benefits of quitting, interrogative cue, self-efficacy cue, and quitline information) based on previous research,^19,21,22,23,24,25^ we found that these elements did not increase smokers’ intentions to quit more than messages featuring a constituent message and an image. It is possible that the image and constituent messages were so impactful that any other message manipulations paled in comparison and thus other message characteristics were not noticed. In an eye-tracking study of similar messages, participants paid the most visual attention to the image, followed by the engagement text, main message text, and the source last.^46^ Indeed, much of the tobacco warning evidence suggests that images significantly augment text-only warnings.^21^ Countries that have implemented graphic tobacco warnings are encouraged to periodically rotate warnings to prevent habituation of the health warning, which suggests the importance of both image and novelty as central to behavior change.^47,48^ It is also possible that some of our message elements, such as engaging information on quitting, may not have influenced quit intentions or other behavioral outcomes but may have influenced secondary outcomes, such as knowledge, self-efficacy to quit, or attitudes toward quitting. Future research could explore these secondary outcomes.

Our study did not find that using the FDA logo enhanced message impact. Presenting the FDA as the source of messages may not have increased intentions to quit because public awareness of the FDA’s role in tobacco regulation is low, and trust in government agencies is also low.^49,50^ It is also possible that few participants in the constituent-plus-engagement condition noticed the FDA source, as was the case in a previous eye-tracking study with similar messages.^46^ Additional research is needed to clarify whether antismoking messages with constituent information substantially benefit from using other message sources (eg, CDC, Surgeon General).

While our study found no significant differences in the number of cigarettes smoked across the 3 study groups, participants reported smoking fewer cigarettes as the number of messages they viewed increased. Participants also reported higher quit intentions at day 32 as the number of messages viewed increased. These findings are consistent with evidence supporting the effect of increased message exposure on promoting smoking cessation^51^ and with a meta-analytic review indicating that repeated exposure promoted greater change in intentions.^52^ More research should be conducted on how dose is associated with smoking cessation outcomes to determine the threshold needed to successfully change behaviors. Although our study did not find significant effects of study condition on our secondary outcomes (ie, putting out and forgoing cigarettes), another RCT study did find a significant change in forgoing behaviors.^17^ This study occurred during a 3-week period, and messages about chemicals in cigarettes were placed on actual cigarette packs. It is possible that this study led to changes in forgoing because individuals who smoked viewed cigarette packs (and messages on those packs) multiple times a day. Therefore, behavioral smoking outcomes may need more intense interventions over time to change.

Findings from the follow-up surveys suggest that quit attempts increased across all conditions between posttest 1 (day 16) and posttest 2 (day 32). It is interesting to note that reported quit attempts continued at the same rate in every study group, absent of any smoking cessation messaging. Research shows that the majority of individuals who smoke (approximately 70%) report wanting to quit and make a number of quit attempts every year^5^ and that it takes an average of almost a dozen quit attempts to succeed.^53^ Therefore, it is reasonable to expect that regardless of the study condition and exposure to messages, participants continued to attempt to quit smoking during the study follow-up period.

### Limitations

Our study has limitations. While our sample included a small pool of previously surveyed individuals who smoke, most came from newly recruited research participants across 46 states, offering some assurance that the sample is more generalizable to adults who continue to smoke. The study design could not differentiate fully between impacts of the constituent message itself compared with the combination of constituent message and image; however, our prior work suggests strongly that the combination of message plus image is more effective than message alone.^16^ We did not biochemically confirm objective measures of smoking, but we did assess self-reported outcomes after each message exposure, which may have led some participants to respond in a socially desirable manner. However, the randomized study design should minimize such concerns, and self-reported intentions^54^ have been shown to be associated with smoking behavior.^55^ Smoking behavior survey items required participants to remember past smoking behaviors during a specific time period, which may have been difficult for some participants to recall. We minimized this limitation by using noon to split the defined time periods (ie, “Yesterday, from the time you woke up until noon, how many cigarettes did you smoke?” and “Yesterday, from noon until you went to sleep, how many cigarettes did you smoke?”). Furthermore, all participants received daily messages, corresponding follow-up surveys, and answered the same questions multiple times over the course of study participation. While the control group received the same questions and a similar intervention, which should protect against testing effects,^56^ it is possible that repeatedly receiving the same survey items multiple times led participants in all conditions to alter their behavior or their responses to items.^57^ The randomized study design equalizes these issues across conditions.

## Conclusions

To our knowledge, this study is the first to longitudinally test a cigarette constituent campaign among a national sample of US adults who currently smoke. The constituent and control messages used in this RCT were developed using established standards for effective tobacco communication.^58^ Our findings suggest that cigarette constituent messages with images increase behavioral intentions to quit smoking among adults who smoke. In addition, as exposure to constituent messages with images increased over time, participants reported smoking fewer cigarettes. These finding hold important implications for the FDA and their education campaigns about cigarette smoke constituents. Using constituent messages with images in communication campaigns may be particularly effective in changing behavioral intentions to quit smoking, a key outcome for adults who smoke.

## Notes

Supplement 1. **Trial Protocol**

Supplement 2. **eAppendix.** Details Regarding Statistical Analysis **eFigure 1. **Messages by Condition **eFigure 2. **Quit Intentions at Pretest, Day 16, and Day 32 **eTable 1. **Quit Intentions at Day 16 and Day 32, Without the Dose Variable **eTable 2. **Secondary Behavioral Outcomes at Day 32, Without the Dose Variable **eTable 3. **Quit Attempts at Day 32, Without the Dose Variable **eTable 4. **Summary Data on Quit Intentions, Secondary Behavioral Outcomes, and Quit Attempts at Pretest, Day 16, and Day 32 **eTable 5. **Quit Attempts at Day 32 **eReferences.**

Supplement 3. **Data Sharing Statement**
