# Supplementary material for: Effect of Cigarette Constituent Messages With Engagement Text on Intention to Quit Smoking Among Adults Who Smoke Cigarettes: A Randomized Clinical Trial
Source: JAMA Netw Open. 2021 Feb 24;4(2):e210045. doi: 10.1001/jamanetworkopen.2021.0045 (PMC7905497; doi:10.1001/jamanetworkopen.2021.0045)
Supplement: Supplement 1. — Trial Protocol [file jamanetwopen-e210045-s001.pdf]

Enhancing Source Credibility in Tobacco Regulatory Communications: Aim 3 RCT

Center for Regulatory Research on Tobacco Communication, Project Credibility (PI: Adam O. Goldstein)

Version 6: Protocol for Publication 8/21/2020

Contents

Administrative Information 4
Trial Registration 4
Funding 4
Roles and Responsibilities 4
Study Methods 5
Population for this study 5
Experimental Conditions 5
Allocation and Blinding 5
Messages 6
Study Flow 7
Days 1-15: Morning Surveys 8
Message Order for Morning Surveys 8
Days 16 and 32: Post-test Surveys 9
Compensation Plan 9
Ethics and Trial Monitoring 10
Data Safety 10
Monitoring 10
Declaration of Interests 10
Dissemination Plan 10
Statistical Analysis Plan 11
Administration Information 11
ClinicalTrials.gov ID 11
SAP Revision History 11
Introduction 11
Background and Rationale 11
Objectives 11
Study Methods 11
Trial Design 11
Randomization 12
Sample Size 12
Sample Size Background: 12
Assumptions for the power calculation: 12
Based on these assumptions: 13
How does this compare to KyungSu's simulations? 13
Recommendation: 13
Framework 14
Statistical Interim Analyses and Stopping Guidance 14
Timing of Final Analysis 14
Timing of Outcome Assessments 14
Statistical Principles 15
Confidence Intervals and P Values 15
Level of statistical significance 15
Description and rationale for any adjustment for multiplicity and, if so detailing how the type 1 error is to be controlled 15
Confidence Intervals to be reported 15
Adherence and Protocol Deviations 15
Analysis Populations 15
Definition of analysis populations 15
Trial Population 16
Screening Data 16

Eligibility ... 16
Recruitment ... 17
Withdrawal/follow-up ... 18
Baseline participant characteristics ... 18
Analysis ... 19
Outcome definitions ... 19
Analysis Methods ... 20
Post test measures ... 20
Daily Questionnaire Measures ... 21
Harms ... 21
Statistical Software ... 21
References ... 21
Appendix A: Study Measures ... 23
Appendix B: Trial Consent Form ... 51

Administrative Information

Trial Registration

Enhancing Source Credibility in Tobacco Regulatory Communications, NCT03339206,
https://clinicaltrials.gov/ct2/show/study/NCT03339206

Registration Date: November 13, 2017

Funding

Research reported in this publication was supported by grant number P50 CA180907 from the National Cancer Institute and the FDA Center for Tobacco Products (CTP). The content is solely the responsibility of the authors and does not necessarily represent the official views of the NIH or the Food and Drug Administration.

Roles and Responsibilities

**Name**	**Role**	**Affiliation**
Adam O. Goldstein	Principal Investigator	Department of Family Medicine, University of North Carolina at Chapel Hill<br>Lineberger Comprehensive Cancer Center, University of North Carolina at Chapel Hill
Kristen L. Jarman	Project Manager	Lineberger Comprehensive Cancer Center, University of North Carolina at Chapel Hill
Sarah D. Kowitt	Doctoral Trainee	Department of Health Behavior, Gillings School of Global Public Health, University of North Carolina at Chapel Hill
Bonnie E. Shook-Sa	Doctoral Trainee	Department of Biostatistics, Gillings School of Global Public Health, University of North Carolina at Chapel Hill
Tara L. Queen	Statistician	Lineberger Comprehensive Cancer Center, University of North Carolina at Chapel Hill
Kyung Su Kim	Statistician	Lineberger Comprehensive Cancer Center, University of North Carolina at Chapel Hill
Leah M. Ranney	Co-Investigator	Department of Family Medicine, University of North Carolina at Chapel Hill
Paschal Sheeran	Co-Investigator	Department of Psychology and Neuroscience, University of North Carolina at Chapel Hill
Seth M. Noar	Co-Investigator	Hussman School of Media and Journalism, University of North Carolina at Chapel Hill

Study Methods

Following a pilot to test intervention feasibility, conduct a randomized controlled trial (RCT) of 800 young adult and adult smokers to test the hypothesis that optimally framed (high source credibility) FDA cigarette constituent messages will increase intentions to quit more than sub-optimally framed (no source attached) cigarette constituent messages or control messages (littering). Messages will be presented in a web-enabled format, derived from cigarette constituent (hereafter referred to as constituent) messages identified in Project 1 of our CRRTC, and framed based on the outcomes from Aim 2 of this project.

Population for this study

1. Age between 18 and 65
2. Current Smoker
3. Not currently enrolled in a smoking cessation program
4. Not currently using pharmacotherapy for smoking cessation
5. Work or home access to the Internet
6. Email account that they regularly use
7. Have not participated in a smoking study, other than phone survey, in last 3 months
8. Able to complete a survey on a computer
9. Able to complete a survey in English
10. Able to complete surveys delivered via email
11. Able to complete 3, 20-minute, surveys
12. Able to complete a 5-min survey each morning for 15 days
13. Lives in the US

Experimental Conditions

- Optimal Group: receive the risk and constituent messages with optimized source depiction and engagement formats.
- Suboptimal Group: receive the risk and constituent messages without source or engagement text.
- Control Group: receive littering messages

Allocation and Blinding

Participants will not be informed specifically about the possible interventions that they may be assigned to. Researchers will not be blinded to the condition that participants had been assigned to, however all outcome measures will be assessed via online survey.

At the end of the baseline survey, survey software will randomly assign participants to one of the three study arms. Participants will have an equal chance of being randomized to each study arm.

Messages

<table>
<tr><th>Constituent Message</th><th>Words (Characters)</th><th>Littering Message</th><th>Words (Characters)</th></tr>
<tr><td>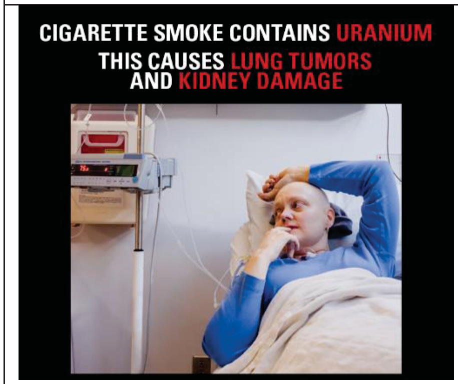
</td><td>11 (76)</td><td>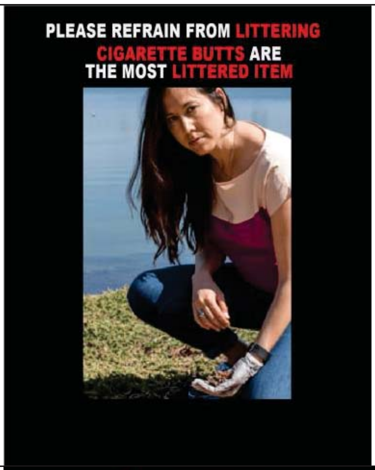
</td><td>11 (74)</td></tr>
<tr><td>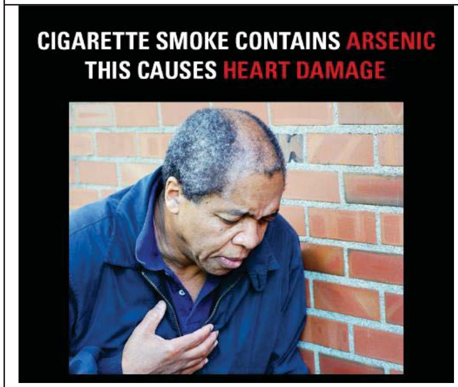
</td><td>8 (59)</td><td>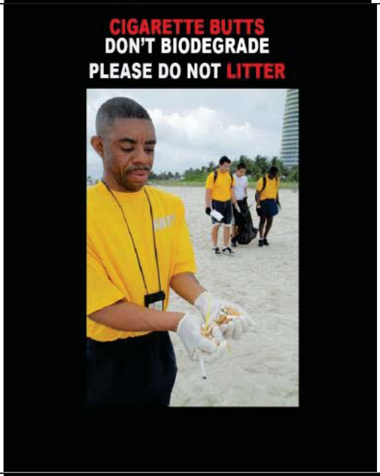
</td><td>8 (55)</td></tr>
<tr><td>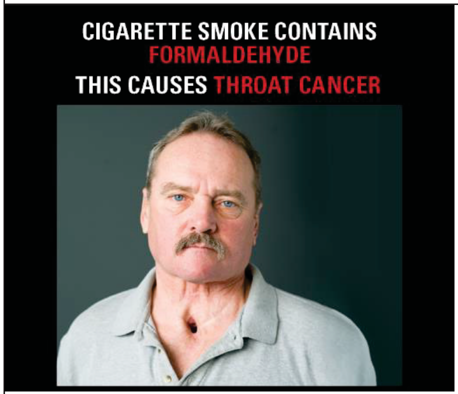
</td><td>8 (65)</td><td>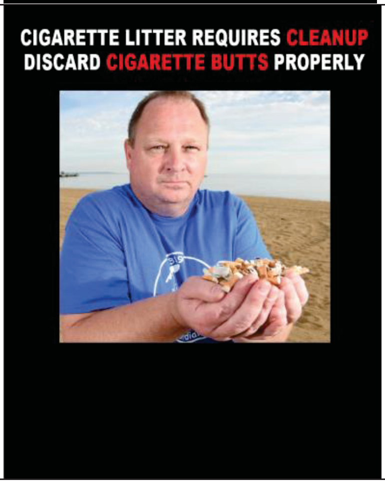
</td><td>8 (68)</td></tr>
</table>

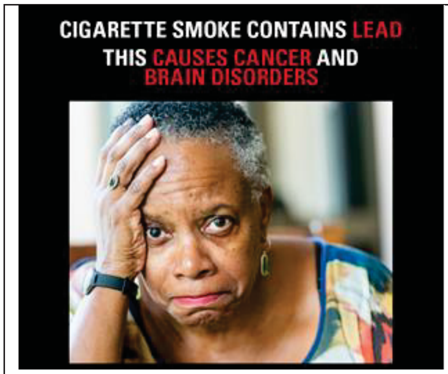	10 (70)	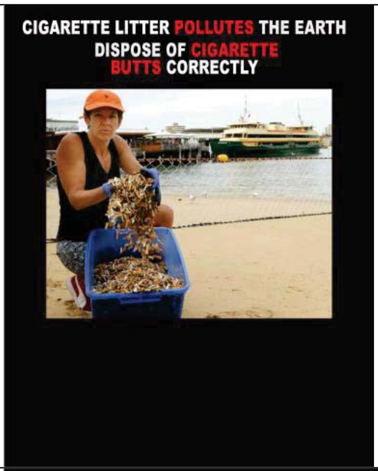	10 (74)
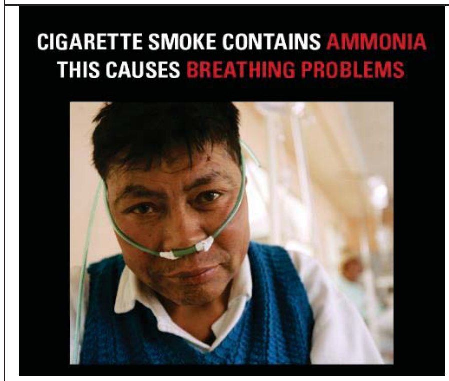	8 (65)	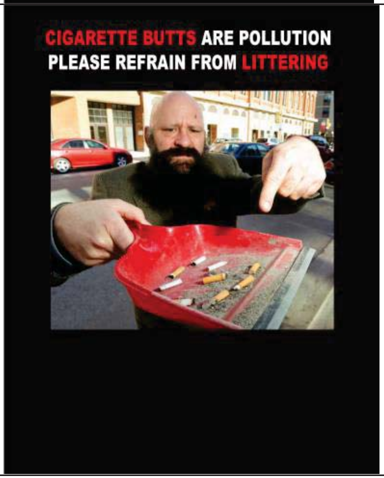	8 (61)

Study Flow

Pilot: Assess whether we can proceed and launch the full study when they have completed post-test 1, allow pilot participants to complete the full study (through post-test 2).

Full Study: Baseline survey, then a survey every morning for 15 days assessing previous day behavior and delivering a message. Follow up with post surveys at day 16 and 32.

Process:

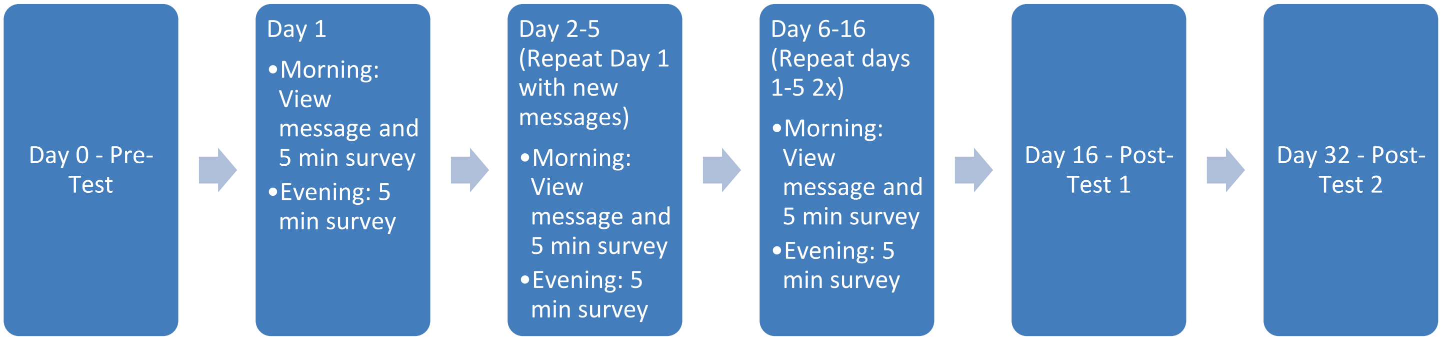

Days 1-15: Morning Surveys

- There are 3 morning surveys, which align with each participant's condition. Participants will stay in their condition throughout the study. Depending on the day, participants will see a different message.
- The morning surveys will be sent out at 7am and will close at 10am. There will be a reminder email automatically sent at 8am if the participant has not yet completed the survey. The surveys will be sent according to each participant's time zone.
- At the end of the morning surveys, participants will see a message saying "You've completed [x] morning survey(s) so far. Keep up the good work!"
- When each survey is emailed out, participants will receive a note that says "If you have any problems, contact tobaccosurvey@unc.edu."

Message Order for Morning Surveys

- In week 1, participants see the following message order: 1-2-5-3-4 (sequence 1)
- In week 2, participants see the following message order: 2-3-1-4-5 (sequence 2)
- In week 3, participants see the following message order: 4-3-5-2-1 (sequence 6)

Day	Message
Day 1	1
Day 8	1
Day 15	1
Day 2	2
Day 6	2
Day 14	2
Day 4	3
Day 7	3
Day 12	3
Day 5	4
Day 9	4
Day 11	4
Day 3	5
Day 10	5
Day 13	5

Day	Message
Day 1	1
Day 2	2
Day 3	5
Day 4	3
Day 5	4
Day 6	2
Day 7	3
Day 8	1
Day 9	4
Day 10	5
Day 11	4
Day 12	3
Day 13	5
Day 14	2
Day 15	1

Conditions 1 and 2:

- Message 1 = Ammonia
- Message 2 = Arsenic
- Message 3 = Formaldehyde
- Message 4 = Lead
- Message 5 = Uranium

Condition 3:

- Message 1 = Bearded man with red trash bin
- Message 2 = Black man with yellow shirt
- Message 3 = White man with blue shirt
- Message 4 = Tan woman with orange hat and lots of cigarette butts in blue bin
- Message 5 = Asian woman with cigarette butts in her hand

Days 16 and 32: Post-test Surveys

- All participants will see the same post-test surveys.
- The post-test surveys will be sent out at 7pm and will close 24 hours later. There will be a reminder email automatically sent at 8pm if the participant has not yet completed the survey. The surveys will be sent according to each participant's time zone.

Compensation Plan

We will pay participants $20 for the initial questionnaire and post-test 1 and post-test 2. We will also pay participants $4 for each morning questionnaire.

Bonus system for daily questionnaire completion:

- Bronze: Total of $10 bonus for at least 9 morning surveys. 1 entry into a drawing for additional money.
- Silver: Total of $20 bonus for at least 12 morning surveys. 2 entries into a drawing for additional money.
- Gold: Total of $30 for completing all 15 morning surveys. 3 entries into a drawing for additional money.

Participants may earn up to $150 for the full study, and will be paid via check.

Drawing for additional money: Three $200 checks will be administered for Bronze, Gold or Silver participants are entered into based on how many daily questionnaires they complete.

Participants will receive a message at the end of each morning survey regarding how many morning surveys they have completed. Participants will be paid after their participation in the study is completed.

Ethics and Trial Monitoring

This trial was approved by UNC's IRB under IRB #17-0610

All protocol changes will be communicated to the study team, and any change in participant interaction will be approved by UNC's IRB.

Data Safety

This study proposes research that has been determined to include Security Level 2 data security requirements. I agree to accept responsibility for managing these risks appropriately in consultation with departmental and/or campus security personnel. The Data Security Requirements addendum can be reviewed here: https://guides.lib.unc.edu/datasecurity/irbis

Monitoring

This trial presents no more than minimal risk to participants, so a data monitoring committee was not necessary. Similarly, the trial did not collect data on harms to participants, or assess interim data during the trial. Similarly, there is no need for ancillary or post-trail care.

Declaration of Interests

The study team declare that they have no competing interests.

Dissemination Plan

The study team plans to disseminate study findings via conference presentation and peer reviewed manuscripts in scientific journals. The study team will also share findings via ClinicalTrials.gov registration.

We will follow authorship guidelines depending on the journal to which manuscripts are submitted.

There are currently no plans to make a participant level dataset public.

Statistical Analysis Plan

Administration Information

ClinicalTrials.gov ID NCT03339206

SAP Revision History

Version Number	Date	Summary of Changes
1	July 24, 2018	SAP Created, agreed to by Adam O. Goldstein (Project PI), Tara L. Queen (Statistical Analyst), KyungSu Kim (Statistical Analyst), and Kristen L. Jarman (Study Coordinator, Document Originator)

Introduction

Background and Rationale

The Family Smoking Prevention and Tobacco Control Act delegates the U.S. Food and Drug Administration (FDA) to communicate the risks of smoking to the public, among other tobacco regulatory responsibilities. Little research exists about how FDA, as a regulatory agency, should develop and deliver these messages, and whether they should include their source information on the ads. This study will assess the impact of cigarette constituent messages with and without FDA source and quit information in a randomized controlled trial. This study will assess the impact of cigarette constituent messages with and without FDA source and quit information in a randomized controlled trial.

Objectives

The investigators hypothesize that cigarette constituent messages will increase intention to quit compared to messages about littering cigarettes (the control). The investigators also hypothesize that constituent messages that include FDA source and quit information will increase intention to quit compared to messages without that information.

Study Methods

Trial Design

Three group parallel trial.

The three conditions are:

- *Cigarette constituent message with FDA source and quit information*: Messages about the chemical constituents of cigarette smoke will include text about chemicals in cigarette smoke and health effects of the chemicals, an image of a person related to the health effect. This arm will also include an FDA logo, and information about the benefits of quitting smoking and the quitline. Each condition has 5 messages that will be repeated 3 times in a counterbalanced order. Study investigators used text developed by investigators associated with our Center for Regulatory Research on Tobacco Communication. Design of these messages was developed by our team.
- *Cigarette constituent message without FDA source or quit information*: Messages about the chemical constituents of cigarette smoke will include text about chemicals in cigarette smoke and health effects of the chemicals, an image of a person related to the health effect. This arm is identical to the arm above, except that it does not include FDA source or quit information. Each condition has 5 messages that will be repeated 3 times in a counterbalanced order. Study investigators used text developed by investigators associated with our Center for Regulatory Research on Tobacco Communication. Design of these messages was developed by our team.
- *Cigarette littering message (Control)*: Messages about littering cigarettes will include text designed to discourage people from littering their cigarette butts, and an image related to the message. Each condition has 5 messages that will be repeated 3 times in a counterbalanced order. Study investigators used text developed by

investigators associated with our Center for Regulatory Research on Tobacco Communication. Design of these messages was developed by our team.

Randomization

At the end of the baseline survey, survey software will randomly assign participants to one of the three study arms. Participants will have an equal chance of being randomized to each study arm.

Sample Size

Sample Size Background:

Our study is examining the difference in quit intention for participants that receive 1) Optimal constituent messages with quitline information, and self-efficacy information, or 2) Sub-optimal constituent messages or 3) Messages about littering cigarette butts.

No studies from the literature that we could find examined difference in quit intention or quitting behaviors by optimal text vs. sub-optimal warning text vs. control in a warning or smoking communication context.

In a meta-analysis by Noar et al. 2014 (Pictorial cigarette pack warnings: a meta-analysis of experimental studies) the effect size between pictorial vs. text only warnings on intention to quit smoking was d=0.54. Other outcomes ranged between effect size d=-0.03 (recall of warning text), and d=1.82 (intention not to start smoking).

Assumptions for the power calculation:

- The smallest effect size between our groups will be between the optimal and sub-optimal group. If we can see a difference between these groups, we will be able to see the larger effects that we expect between the other groups.
- We will be using a continuous measure of quit intention (or treating quit intention as a continuous variable, and collecting it as a categorical response).
- The effect size on quit intention is likely to be smaller for added self efficacy and quit line text than for added pictorial images.
- To be conservative, we will use a one time measure to calculate power, repeated measures would add power to this analysis (decrease necessary sample size).
- We will use the d statistic to see what group size would have to be enrolled, and compare two means (optimal and sub-optimal) with a t-test

Based on these assumptions:

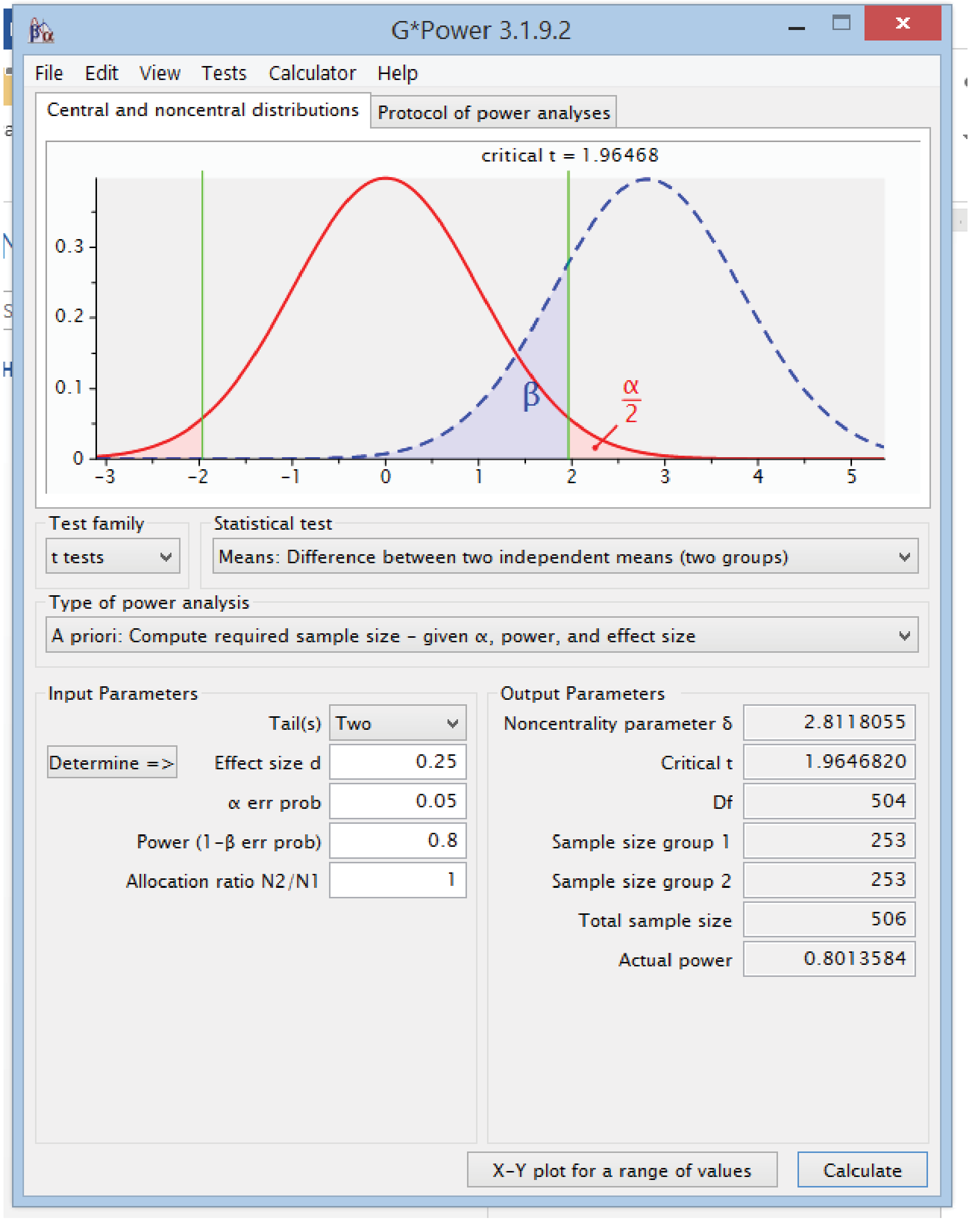

The power calculation above is for a smaller effect size than the Noar 2016 paper, d=0.25. If we enroll 253 per group, we will have 80% power to detect a small difference between the optimal and sub-optimal group.

For our purposes, that would mean enrolling a total of at least 759 people.

How does this compare to KyungSu's simulations?

Based on Kyung Su's simulations, if we use 3 time points, and enroll around 750 people, we should be able to see an effect size of 0.35. This simulation is also very dependent on the assumptions that we started with.

Recommendation:

To be even more conservative, I would like to enroll at least 800 participants into our study to have enough power to see a d=0.25 difference between the optimal and sub-optimal groups.

Framework

Superiority – standard hypothesis testing framework

Statistical Interim Analyses and Stopping Guidance

NA, this study is no more than minimal risk to participants and does not pose a risk to their health, so we did not carry out any interim analyses or set guidelines for stopping the trial early.

Timing of Final Analysis

Final analysis will take place after all data collection for the RCT is complete.

Timing of Outcome Assessments

Time	Pre-Test	Morning	Post-Test 1	Post-Test 2
Constructs	• Sex, Sexuality<br>• Hispanic Origin, Race<br>• Address<br>• Education<br>• Age<br>• Health<br>• Household Size<br>• Income (above or below FPL)<br>• Trust in FDA and CDC<br>• Nicotine Dependence (FTND)<br>• Previous Quit Attempts<br>• Smoking Status<br>• Quit Intentions<br>• Littering Intention<br>• Self Efficacy<br>• Response Efficacy<br>• Positive and Negative Thoughts (Freq, Degree)<br>• Current OTP Use<br>• Knowledge of HE and Constituents<br>• Tripartite Risk<br>• Mental Health (K6)<br>• Study Information<br>• **All Previous Day Behavior Qs**	Previous day behaviors:<br>• Littering on the ground<br>• Littering from Car<br>• Forgoing<br>• Butting out<br>• Number of Cigarettes<br>Cognitive Questions:<br>• Thinking about chemicals<br>• Thinking about risks<br>• Thinking about littering<br>• Thinking about quitting<br>• **Message**<br>• Negative Affect<br>• Message Credibility<br>• Perceived Effectiveness	• Nicotine Dependence (FTND)<br>• Quit Attempts<br>• Smoking Status<br>• Quitline Awareness<br>• Quitline Use<br>• Quit Intentions<br>• Quitline Intention<br>• Littering Intention<br>• Self Efficacy<br>• Response Efficacy<br>• Positive and Negative Thoughts (Freq, Degree)<br>• Current OTP Use<br>• Knowledge of HE and Constituents<br>• Tripartite Risk<br>• Credibility of FDA<br>• Mental Health (K6)<br>• Action Expectancies<br>• Reactance<br>• Talking about messages<br>• **All Previous Day Behavior Qs**<br>• Quality Assurance Questions	• Nicotine Dependence (FTND)<br>• Quit Attempts<br>• Smoking Status<br>• Quitline Use<br>• Quit Intentions<br>• Quitline Intention<br>• Littering Intention<br>• Self Efficacy<br>• Response Efficacy<br>• Positive and Negative Thoughts (Freq, Degree)<br>• Current OTP Use<br>• Knowledge of HE and Constituents<br>• Tripartite Risk<br>• Mental Health (K6)<br>• Action Expectancies<br>• **All Previous Day Behavior Qs**

Statistical Principles

Confidence Intervals and P Values

Level of statistical significance

α=0.05

Description and rationale for any adjustment for multiplicity and, if so detailing how the type 1 error is to be controlled

NA

Confidence Intervals to be reported

95% CI

Adherence and Protocol Deviations

Definition of adherence to the intervention and how this is assessed including extent of exposure

Compliance with the protocol is assessed as follows:

- Completion of daily surveys, during which participants view intervention stimuli. % Compliance = number of daily surveys completed / 15 (number of surveys supposed to be completed)*100%
- Completion of post test surveys at post 1 and post 2

Description of how adherence to the intervention will be presented

- Adherence for the daily surveys will be presented with completion % as well as the mean number complete by treatment group
- Completion of post test surveys will be presented by % completing each by treatment group

Definition of protocol deviations for the trial

- Minor protocol deviation: change in eligibility (ex: turning 66) between screening and baseline
- Minor protocol deviation: completing the wrong sequence of message days due to survey signal error
- Major protocol deviation: No protocol deviations in this trial will impact the safety or physical or mental integrity of the participants in the trial. A slightly older participant, or a participant that receives a slightly different number of messages will both be included as covariates during analysis, so they will not impact the scientific validity of the trial. Therefore, we do not classify any protocol deviations as 'major'

Description of which protocol deviations will be summarized

- Protocol deviations will not be summarized overall, but data related to the deviations will be included in manuscripts. For example, we will not say X participants were older than 65, but will include mean age in a demographic table by treatment group. Similarly, we will not include how many people completed an extra 'day' of the protocol, but we will report the mean number of days participants completed by treatment group.

Analysis Populations

Definition of analysis populations

Outcomes assessed only at post test (including quit intentions) will be analyzed using multiple imputation such that everyone who was randomized will be included in the analysis, and those with missing outcome data will have their outcomes imputed.

For outcomes that were measured daily, all participants that completed at least one of the daily surveys will be included in the planned repeated measure models.

Secondary outcomes may be analyzed using complete case analysis, without imputation.

Trial Population

Screening Data

Screening data will be provided based on the CONSORT guidelines, and CONSORT flow chart will be included with publications.

Eligibility

Minimum Age: 18 Years

Maximum Age: 65 Years

Sex: All

Gender Based: No

Inclusion Criteria:

- Have smoked at least 100 cigarettes in his or her lifetime
- Currently smoke cigarettes every day or some days
- Work or home access to the internet
- Email account that is regularly used
- Lives in the US
- Comfortable taking a survey in English
- Able to complete a survey on a computer
- Able to complete surveys delivered via email
- Able to complete 3, 20 minute surveys during the study
- Able to complete surveys in the morning for 15 days

Exclusion Criteria:

- Currently using pharmacotherapy, a quitline, or a quit smoking program or support group for smoking cessation
- Participated in a research study about smoking cigarettes or using other tobacco products in the last 3 months

Recruitment

Recruitment data will be provided based on the CONSORT guidelines. The following CONSORT chart will be used as a template for reporting recruitment.

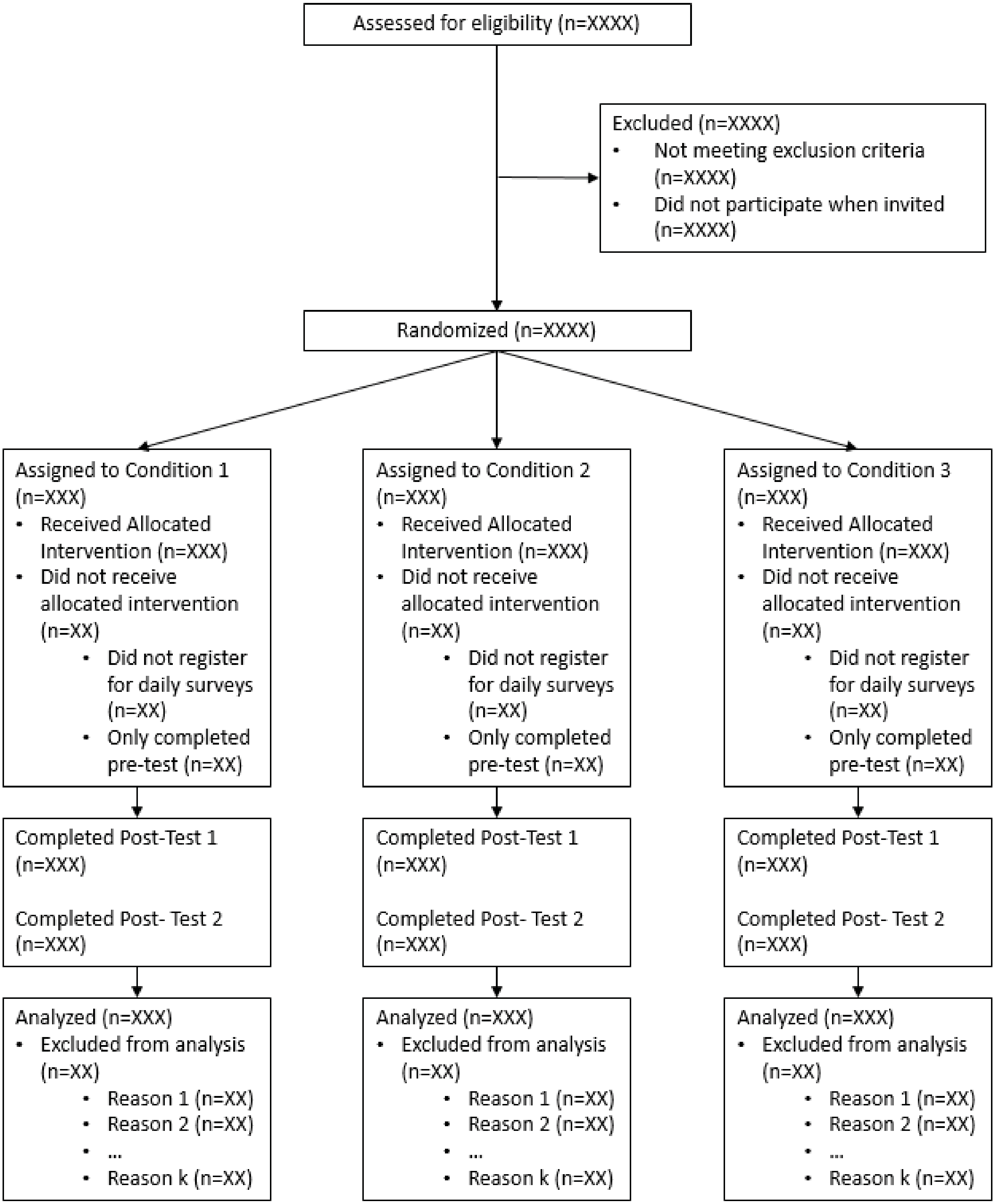

Withdrawal/follow-up

Level of withdrawal

- Data collection and the intervention occur using the same surveys, when a participant withdraws from data collection, they are withdrawing from follow up and the intervention.

Timing of withdrawal/lost to follow-up data

- Timing of withdrawal will be presented by treatment group, and categorized into each of the following timepoints:
    - After baseline but before beginning intervention (before completing first daily survey)
    - During intervention (days 1-15)
    - During post-test follow up period (days 16-32)
    - After post-test follow up period (after day 32)

Reasons and details of how withdrawal/lost to follow-up data will be presented

- Numbers (with reasons) of loss to follow up and withdrawal over the course of the trial will be summarized by treatment group

Baseline participant characteristics

List of baseline characteristics to be summarized

See Draft Table 1 below.

Details of how baseline characteristics will be descriptively summarized

Baseline characteristics will be descriptively summarized as show in Draft Table 1.S

Draft Table 1. Baseline Sample Characteristics

Sample Characteristics	Condition 1 (n=XXX)		Condition 2 (n=XXX)		Condition 3 (n=XXX)		Total Sample (n=XXX)	
	% or mean	95% CI	% or mean	95% CI	% or mean	95% CI	% or mean	95% CI
Gender								
Male								
Female								
Age, years								
Race								
White								
Black or African American								
Other Race								
Ethnicity								
Latino/Hispanic								
Non-Latino/Hispanic								
Education								
<High School (HS)								
G12 or GED, HS Diploma								
Some College								

	Associate's degree								
	Bachelor's degree								
	Graduate or professional degree								
Poverty Status									
	Below Poverty Line								
	Above Poverty Line								
Sexual Orientation									
	Straight or heterosexual								
	Gay, lesbian, or bisexual								
Current Cigarette Smoking									
	Current smoker								
	Nonsmoker								
Fagerstrom Nicotine Dependence Score (FTND)									
Trust in FDA									
Number of Times Participants Viewed a Message During Follow Up									

To update Table 1 see ‘Table 1 V1.xlsx’ in Analysis Plan folder

Analysis

Outcome definitions

- Quit intentions [Time Frame: day 16] PRIMARY OUTCOME
    - 3 item measure with high reliability, average of the 3 items to create a score range 1-4 (Brewer et al., 2016, 2018; Klein, Zajac, & Monin, 2009)
    - We will compare the differences between pre and post
- Number of cigarettes smoked each day [Time Frame: Measured daily on days 1-15]
    - Continuous, Modified from FTND (Heatherton, Kozlowski, Frecker, & Fagerström, 1991; Storr, Reboussin, & Anthony, 2005)
- Number of cigarettes forgone each day [Time Frame: Measured daily on days 1-15]
    - Continuous, adapted from Brewer 2016 (Brewer et al., 2016)
    - May be zero inflated, if yes, may choose to dichotomize to 0/1
- Number of cigarettes butted out each day [Time Frame: Measured daily on days 1-15]
    - Continuous, adapted from Brewer 2016 (Brewer et al., 2016)
    - May be zero inflated, if yes, may choose to dichotomize to 0/1
- Quit attempts during the study [Time Frame: Measured on days 16 and 32]
    - Adaptation from Fagan (Fagan et al., 2007)
    - May be zero inflated, if yes, may choose to dichotomize to 0/1
- Awareness of quitline [Time Frame: Measured on day 16]
- Recall of quitline phone number [Time Frame: Measured on day 16]
- Use of the quitline during the study [Time Frame: Measured on days 16 and 32]
- Intention to use the quitline [Time Frame: Measured on days 16 and 32]
- Quit intentions [Time Frame: Measured on day 32]
    - 3 item measure with high reliability, average of the 3 items to create a score range 1-4 (Brewer et al., 2016, 2018; Klein et al., 2009)
    - We will compare the differences between pre and post
- Self Efficacy [Time Frame: Measured on days 16 and 32]

    - 1 item (“IARC HANDBOOKS OF CANCER PREVENTION,” n.d.; Thrasher, Swayampakala, Borland, et al., 2016; Thrasher, Swayampakala, Cummings, et al., 2016)
- Response Efficacy [Time Frame: Measured on days 16 and 32]
    - 1 item (Thrasher, Swayampakala, Cummings, et al., 2016)

Analysis Methods

Analysis methods will depend on the time point that the outcome was measured. Post test measures will be analyzed differently than measures from the daily surveys, as outlined below.

Post test measures

What analysis method will be used and how treatment effects will be presented

Linear regression for continuous outcomes, logistic for dichotomous.

Any adjustment for covariates

Covariates included for the imputation (in addition to treatment group):

- Dose of ads (# of times participants viewed a message)
- Gender (Male, Female, Other) (The GenIUSS Group, 2014)
- Age (continuous)
- Poverty Status (Above or Below Poverty Line)(Brewer et al., 2018; Department of Health and Human Services, 2017)
- Education (6 categories, same as phone survey)
- FTND Sum Using scoring from Heatherton et al. 1991 (Fagerstrom Test for Nicotine Dependence)(Fagerstrom, Heatherton, & Kozlowski, 1990; Heatherton et al., 1991)

Covariates included in the analysis model (in addition to treatment group):

- Dose of ads (# of times participants viewed a message)

Methods used for assumptions to be checked for statistical methods

For continuous outcomes, we assume the distribution of dependent variable given a predictor variable (intervention) is 1) independent 2) normality 3) constant variance (homogeneity of variance). We can check normality with Q-Q plot of the residual or perform Wilk-Shapiro test and use a scatter plot of residual on predictor variable (intervention) to check independence and constant variance.

Details of alternative methods to be used if distributional assumptions do not hold (eg normality, proportional hazards, etc)

If we have a violation for normality assumption, we can have two options: 1) transform our data so that the shape of our response variable become normally distributed or 2) choose the non-parametric method like Kruskal-Wallis which does not require the assumption of normality. The ANOVA can be considered a robust test against the normality assumption though. If the assumption of homogeneity of variances has been violated, we can use Welch or Brown and Forsythe test. Even though we alternatively perform a Kruskal-Wallis test, in most cases, Welch test is best. We don’t expect to have dependency issues in our data.

Any planned sensitivity analyses for each outcome where applicable

Sensitivity analyses may be carried out using a ‘complete case analysis’ framework

Any planned subgroup analyses for each outcome including how subgroups are defined

None a priori

Missing Data

Multiple imputation will be used so that everyone who was randomized to an intervention will be included in the primary analysis

Additional Analyses

None a priori

Daily Questionnaire Measures

What analysis method will be used and how treatment effects will be presented
Mixed models to account for repeated measures of the same participant.

Any adjustment for covariates
Covariates included in the analysis model (in addition to treatment group):

- Dose of ads (# of times participants viewed a message)

Methods used for assumptions to be checked for statistical methods
Linear mixed model is an extremely flexible for modeling continuous outcomes. It turns out that linear mixed models are robust to violations of some of their assumptions. The followings are the assumptions for linear mixed model: 1) Independent (The outcomes of different people are assumed to be statistically independent of each other.) Note: the outcomes within a person are correlated. 2) Normality (random effects are assumed to be normally distributed with mean zero and the residuals are normally distributed.)

Details of alternative methods to be used if distributional assumptions do not hold (eg normality, proportional hazards, etc)
Because mixed models are robust to violations of some of their assumptions, we do not anticipate needing to use an alternative method.

Any planned sensitivity analyses for each outcome where applicable
None a priori

Any planned subgroup analyses for each outcome including how subgroups are defined
None a priori

Missing Data
Mixed models are appropriate to use and robust to missing data, so we will be able to include everyone who completed at least 1 daily questionnaire.

Additional Analyses
None a priori

Harms

Adverse events were not expected in this trial due to the minimal risks to participants, and no threat to participant health, so this section is not applicable.

Statistical Software

Statistical analyses will be conducted in SAS 9.4 (Cary, NC)

References

Final Dataset: rct_combined_07232018 [location removed]

Data Cleaning Syntax: 01_Create_Analytic_Files.sas [location removed]

Study Protocol: RCT_Protocol_V5 [location removed]

Analysis plan compiled according to “Guidelines for the Content of Statistical Analysis Plans in Clinical Trials” (Gamble et al., 2017)

Brewer, N. T., Hall, M. G., Noar, S. M., Parada, H., Stein-Seroussi, A., Bach, L. E., … Ribisl, K. M. (2016). Effect of Pictorial Cigarette Pack Warnings on Changes in Smoking Behavior: A Randomized Clinical Trial. *JAMA Internal Medicine*, *176*(7), 905–912. https://doi.org/10.1001/jamainternmed.2016.2621

Brewer, N. T., Jeong, M., Mendel, J. R., Hall, M. G., Zhang, D., Parada, H., … Ribisl, K. M. (2018). Cigarette pack messages about toxic chemicals: a randomised clinical trial. *Tobacco Control*, tobaccocontrol-2017-054112. https://doi.org/10.1136/tobaccocontrol-2017-054112

Department of Health and Human Services. (2017). *2017 Annual Update of the HHS Poverty Guidelines*. *Federal Register* (Vol. 82). Retrieved from https://www.gpo.gov/fdsys/pkg/FR-2017-01-31/pdf/2017-02076.pdf

Fagan, P., Augustson, E., Backinger, C. L., O'Connell, M. E., Vollinger, R. E., Kaufman, A., … Gibson, J. T. (2007). Quit attempts and intention to quit cigarette smoking among young adults in the United States. *American Journal of Public Health*, *97*(8), 1412–1420. https://doi.org/10.2105/AJPH.2006.103697

Fagerstrom, K. O., Heatherton, T. F., & Kozlowski, L. T. (1990). Nicotine addiction and its assessment. *Ear, Nose, & Throat Journal*, *69*(11), 763–765. Retrieved from http://www.ncbi.nlm.nih.gov/pubmed/2276350

Gamble, C., Krishan, A., Stocken, D., Lewis, S., Juszczak, E., Doré, C., … Loder, E. (2017). Guidelines for the Content of Statistical Analysis Plans in Clinical Trials. *JAMA*, *318*(23), 2337. https://doi.org/10.1001/jama.2017.18556

Heatherton, T. F., Kozlowski, L. T., Frecker, R. C., & Fagerström, K. O. (1991). The Fagerström Test for Nicotine Dependence: a revision of the Fagerström Tolerance Questionnaire. *British Journal of Addiction*, *86*(9), 1119–1127. Retrieved from http://www.ncbi.nlm.nih.gov/pubmed/1932883

IARC HANDBOOKS OF CANCER PREVENTION. (n.d.). *International Agency for Research on Cancer World Health Organization*, *12*. Retrieved from http://www.iarc.fr/en/publications/pdfs-online/prev/handbook12/Tobacco_vol12_cover.pdf

Klein, W. M. P., Zajac, L. E., & Monin, M. M. (2009). Worry as a Moderator of the Association Between Risk Perceptions and Quitting Intentions in Young Adult and Adult Smokers. *Annals of Behavioral Medicine*, *38*(3), 256–261. https://doi.org/10.1007/s12160-009-9143-2

Storr, C. L., Reboussin, B. A., & Anthony, J. C. (2005). The Fagerström test for nicotine dependence: A comparison of standard scoring and latent class analysis approaches. *Drug and Alcohol Dependence*, *80*(2), 241–250. https://doi.org/10.1016/j.drugalcdep.2004.04.021

The GenIUSS Group. (2014). *Best Practices for Asking Questions to Idenfity Transgender and Other Gender Minority Respondents on Population-Based Surveys*. Retrieved from https://williamsinstitute.law.ucla.edu/wp-content/uploads/geniuss-report-sep-2014.pdf

Thrasher, J. F., Swayampakala, K., Borland, R., Nagelhout, G., Yong, H.-H., Hammond, D., … Hardin, J. (2016). Influences of Self-Efficacy, Response Efficacy, and Reactance on Responses to Cigarette Health Warnings: A Longitudinal Study of Adult Smokers in Australia and Canada. *Health Communication*, *31*(12), 1517–1526. https://doi.org/10.1080/10410236.2015.1089456

Thrasher, J. F., Swayampakala, K., Cummings, K. M., Hammond, D., Anshari, D., Krugman, D. M., & Hardin, J. W. (2016). Cigarette package inserts can promote efficacy beliefs and sustained smoking cessation attempts: A longitudinal assessment of an innovative policy in Canada. *Preventive Medicine*, *88*, 59–65. https://doi.org/10.1016/j.ypmed.2016.03.006

Appendix A: Study Measures

Constructs by Timeframe

Time	Pre-Test	Morning	Post-Test 1	Post-Test 2
Constructs	• Sex, Sexuality<br>• Hispanic Origin, Race<br>• Address<br>• Education<br>• Age<br>• Health<br>• Household Size<br>• Income (above or below FPL)<br>• Trust in FDA and CDC<br>• Nicotine Dependence (FTND)<br>• Previous Quit Attempts<br>• Smoking Status<br>• Quit Intentions<br>• Littering Intention<br>• Self Efficacy<br>• Response Efficacy<br>• Positive and Negative Thoughts (Freq, Degree)<br>• Current OTP Use<br>• Knowledge of HE and Constituents<br>• Tripartite RiskMental Health (K6)<br>• Study Information<br>• **All Previous Day Behavior Qs**	Previous day behaviors:<br>• Littering on the ground<br>• Littering from Car<br>• Forgoing<br>• Butting out<br>• Number of Cigarettes<br>Cognitive Questions:<br>• Thinking about chemicals<br>• Thinking about risks<br>• Thinking about littering<br>• Thinking about quitting<br>• **Message**<br>• Negative Affect<br>• Message Credibility<br>• Perceived Effectiveness	• Nicotine Dependence (FTND)<br>• Quit Attempts<br>• Smoking Status<br>• Quitline Awareness<br>• Quitline Use<br>• Quit Intentions<br>• Quitline Intention<br>• Littering Intention<br>• Self Efficacy<br>• Response Efficacy<br>• Positive and Negative Thoughts (Freq, Degree)<br>• Current OTP Use<br>• Knowledge of HE and Constituents<br>• Tripartite Risk<br>• Credibility of FDA<br>• Mental Health (K6)<br>• Action Expectancies<br>• Reactance<br>• Talking about messages<br>• **All Previous Day Behavior Qs**<br>• Quality Assurance Questions	• Nicotine Dependence (FTND)<br>• Quit Attempts<br>• Smoking Status<br>• Quitline Use<br>• Quit Intentions<br>• Quitline Intention<br>• Littering Intention<br>• Self Efficacy<br>• Response Efficacy<br>• Positive and Negative Thoughts (Freq, Degree)<br>• Current OTP Use<br>• Knowledge of HE and Constituents<br>• Tripartite Risk<br>• Mental Health (K6)<br>• Action Expectancies<br>• **All Previous Day Behavior Qs**

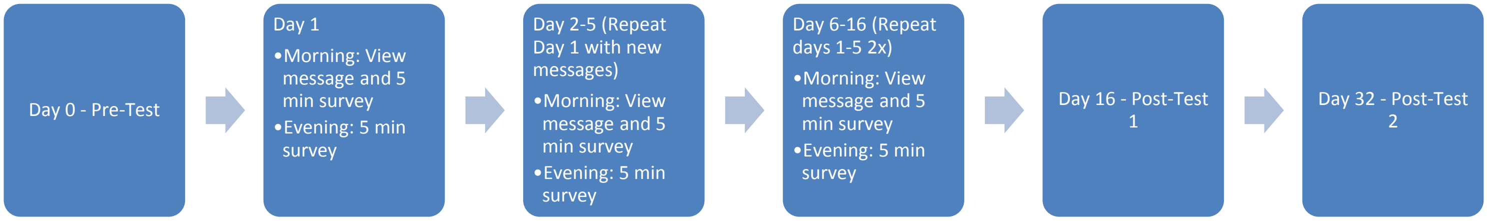

Pre, Post 1, Post 2

Construct (Source)	Question Text	Response Option	Pretest Var Name	Post 1 Var Name	Post 2 Var Name
Instruction	Please read the consent form on the next page to learn more about our research study and what we are asking participants to do.		Instruction		
Post 1 Instruction	You have now finished the part of the study with daily surveys.<br><br>This slightly longer survey should take you less than 20 minutes to complete. If you complete the survey, you will be paid an additional $20 in your incentive check.			Post1_Inst	
Post 2 Instruction	This will be the last survey for this study. Thank you for your participation!<br><br>This survey should take you less than 20 minutes to complete. If you complete the survey, you will be paid an additional $20 in your incentive check.				Post2_Inst
PAGE BREAK					

Construct (Source)	Question Text	Response Option	Pretest Var Name	Post 1 Var Name	Post 2 Var Name
Consent Form	[Include current consent form with agreement to participate]		ConsentForm1, ConsentForm_GraphicConsentForm2, ConsentForm3		
PAGE BREAK					
Sex1[1]	What sex were you assigned at birth, on your original birth certificate? Was it…	1 = Male<br>2 = Female	Sex1		
Sex2[1]	Do you describe yourself as…	1= Male<br>2= Female<br>3= Transgender<br>4= Other (specify_______________)	Sex2, Sex2_Text		
Sexual Orientation[2,3]	Do you consider yourself to be…	1= Straight or heterosexual<br>2= Gay or lesbian<br>3= Bisexual<br>4= OTHER (specify_______________)	Sexuality, Sexuality_Text		
Page Break					
Hispanic Origin[3]	Are you of Hispanic, Latino, or Spanish origin?	0= No<br>1 = Yes	Hisp		
Race[3]	Which one of these groups would you say best represents your race?	1=White<br>2=Black or African American<br>3=American Indian or Alaska Native<br>4=Asian<br>5=Pacific Islander<br>6=Other	Race		
Page Break					

Construct (Source)	Question Text	Response Option	Pretest Var Name	Post 1 Var Name	Post 2 Var Name
Instruction to provide name and address	Please provide your mailing address below. This is where we will send your incentive check at the end of the study.		Instr3		
First Name	First Name	Free Response	FirstName		
Last Name	Last Name	Free Response	LastName		
Address Line 1	Street Number, Street Name	Free Response (Validity Check)	Addr1		
Address Line 2	Apartment Number, Optional Address Line 2	Free Response (Validity Check, Optional Response)	Addr2		
City	City	Free Response (Validity Check)	City		
Zip	Zip Code	Free Response	Zip		
State	State	Dropdown list<br>1= ALABAMA<br>2= ALASKA<br>3= ARIZONA<br>4= ARKANSAS<br>5= CALIFORNIA<br>6= COLORADO<br>7= CONNECTICUT<br>8= DELAWARE<br>9= DISTRICT OF COLUMBIA<br>10= FLORIDA<br>11= GEORGIA<br>12= HAWAII<br>13= IDAHO<br>14= ILLINOIS<br>15= INDIANA<br>16= IOWA<br>17= KANSAS<br>18= KENTUCKY<br>19= LOUISIANA<br>20= MAINE<br>21= MARYLAND<br>22= MASSACHUSETTS<br>23= MICHIGAN<br>24= MINNESOTA<br>25= MISSISSIPPI	State_0		

Construct (Source)	Question Text	Response Option	Pretest Var Name	Post 1 Var Name	Post 2 Var Name
		26= MISSOURI<br>27= MONTANA<br>28= NEBRASKA<br>29= NEVADA<br>30= NEW HAMPSHIRE<br>31= NEW JERSEY<br>32= NEW MEXICO<br>33= NEW YORK<br>34= NORTH CAROLINA<br>35= NORTH DAKOTA<br>36= OHIO<br>37= OKLAHOMA<br>38= OREGON<br>39= PENNSYLVANIA<br>(40=Puerto Rico* Option not displayed, included to align with screener for phone survey participants)<br>41= RHODE ISLAND<br>42= SOUTH CAROLINA<br>43= SOUTH DAKOTA<br>44= TENNESSEE<br>45= TEXAS<br>46= UTAH<br>47= VERMONT<br>48= VIRGINIA<br>49= WASHINGTON<br>50= WEST VIRGINIA<br>51= WISCONSIN<br>52= WYOMING			
PAGE BREAK					
Time Zone	What time zone are you in?	-5=Eastern Standard Time (Ex: New York)<br>-6=Central Standard Time (Ex: Chicago)<br>-7=Mountain Standard Time (Ex: Salt Lake City)<br>-8=Pacific Standard Time (Ex: Los Angeles)<br>-9=Alaska Standard Time (Ex: Anchorage)<br>-10=Hawaii-Aleutian Standard Time (Ex: Honolulu)	TimeZone		
PAGE BREAK					
Cell Phone Number	Do you have a cell phone?	1=Yes<br>0=No	Cell_YN		
PAGE BREAK					

Construct (Source)	Question Text	Response Option	Pretest Var Name	Post 1 Var Name	Post 2 Var Name
Cell Phone Number	Cell Phone Number	Free Response (Validity Check)	Phone_Number		
Page Break					
Education[3]	What is the highest degree or level of school you have completed?	[0= NO SCHOOLING COMPLETED<br>1= NURSERY SCHOOL TO 4TH GRADE<br>2= 5TH OR 6TH GRADE<br>3= 7TH OR 8TH GRADE<br>4= 9TH GRADE<br>5= 10TH GRADE<br>6= 11TH GRADE<br>7= 12TH GRADE - NO DIPLOMA<br>8= HIGH SCHOOL GRADUATE - high school diploma or the equivalent (for example: GED)<br>9= SOME COLLEGE CREDIT, BUT LESS THAN 1 YEAR<br>10= 1 OR MORE YEARS OF COLLEGE, NO DEGREE<br>11= ASSOCIATE'S DEGREE (FOR EXAMPLE: AA, AS)<br>12= BACHELOR'S DEGREE (FOR EXAMPLE: BA, AB, BS)<br>13= MASTER'S DEGREE (FOR EXAMPLE: MA, MS, MEng, MEd, MSW, MBA)<br>14= PROFESSIONAL DEGREE (FOR EXAMPLE: MD, DDS, DVM, LLB, JD)<br>15= DOCTORATE DEGREE (FOR EXAMPLE: PhD, EdD)]	Edu		

Construct (Source)	Question Text	Response Option	Pretest Var Name	Post 1 Var Name	Post 2 Var Name
Page Break					
Age	How old are you?	Free response (Must be 3 or fewer numbers, between 18-120)	Age		
General Health[3]	Would you say that in general your health is:	5= Excellent<br>4= Very Good<br>3= Good<br>2= Fair<br>1= Poor	Health		
Household[4]	How many people are in your household, including you?	# of people [restricted to 1-10]	Household		
Income[4,5]	[Ask only if Household = 1, else skip to FTND 1]<br><br>Which of the following categories best describes your total household income in the last 12 months?	1=Less than $12,060<br>2=Between $12,060 and $18,089<br>3=Between $18,090 and $24,119<br>4=Between $24,120 and $30,149<br>5=Between $30,150 and $36,179<br>6=$36,180 or more	Income_1		
	[Ask only if Household = 2, else skip to FTND 1]<br><br>Which of the following categories best describes your total household income in the last 12 months?	1=Less than $16,240<br>2=Between $16,240 and $24,359<br>3=Between $24,360 and $32,479<br>4=Between $32,480 and $40,599<br>5=Between $40,600 and $48,719<br>6=$48,720 or more	Income_2		
	[Ask only if Household = 3, else skip to FTND 1]<br><br>Which of the following categories best describes your total household income in the last 12 months?	1=Less than $20,420<br>2=Between $20,420 and $30,629<br>3=Between $30,630 and $40,839<br>4=Between $40,840 and $51,049<br>5= Between $51,050 and $61,259<br>6=$61,260 or more	Income_3		
	[Ask only if Household = 4, else skip to FTND 1]	1=Less than $24,600<br>2=Between $24,600 and $36,899<br>3=Between $36,900 and $49,199	Income_4		

Construct (Source)	Question Text	Response Option	Pretest Var Name	Post 1 Var Name	Post 2 Var Name
	Which of the following categories best describes your total household income in the last 12 months?	4=Between $49,200 and $61,499<br>5=Between $61,500 and $73,799<br>6=$73,800 or more			
	[Ask only if Household = 5, else skip to FTND 1]<br><br>Which of the following categories best describes your total household income in the last 12 months?	1=Less than $28,780<br>2=Between $28,780 and $43,169<br>3=Between $43,170 and $57,559<br>4=Between $57,560 and $71,949<br>5=Between $71,950 and $86,339<br>6=$86,430 or more	Income_5		
	[Ask only if Household = 6, else skip to FTND 1]<br><br>Which of the following categories best describes your total household income in the last 12 months?	1=Less than $32,960<br>2=Between $32,960 and $49,439<br>3=Between $49,440 and $65,919<br>4=Between $65,920 and $82,399<br>5=Between $82,400 and $98,879<br>6=$98,880 or more	Income_6		
	[Ask only if Household = 7, else skip to FTND 1]<br><br>Which of the following categories best describes your total household income in the last 12 months?	1=Less than$37,140<br>2=Between $37,140 and $55,709<br>3=Between $55,710 and $74,279<br>4=Between $74,280 and $92,849<br>5= Between $92,850 and $111,419<br>6=$111,420 or more	Income_7		
	[Ask only if Household = 8, else skip to FTND 1]<br><br>Which of the following categories best describes your total household income in the last 12 months?	1=Less than $41,320<br>2=Between $41,320 and $61,979<br>3=Between $61,980 and $82,639<br>4=Between $82,640 and $103,299<br>5=Between $103,300 and $123,959<br>6=$123,960 or more	Income_8		
	[Ask only if Household = 9, else skip to FTND 1]	1=Less than $45,500<br>2=Between $45,500 and $68,249<br>3=Between $68,250 and $90,999	Income_9		

Construct (Source)	Question Text	Response Option	Pretest Var Name	Post 1 Var Name	Post 2 Var Name
	Which of the following categories best describes your total household income in the last 12 months?	4=Between $91,000 and $113,749<br>5=Between $113,750 and $136,549<br>6=$136,500 or more			
	[Ask only if Household = 10, else skip to FTND 1]<br><br>Which of the following categories best describes your total household income in the last 12 months?	1=Less than $49,680<br>2=Between $49,680 and $74,519<br>3=Between $74,520 and $99,359<br>4=Between $99,360 and $124,199<br>5=Between $124,200 and $149,039<br>6=$149,040 or more	Income_10		
Page Break					
Trust in Gov[3,6]	How much trust do you have in the Federal government?	5= A great deal<br>4= A fair amount<br>2= Not very much<br>1= None at all, or<br>3= No opinion	TrustGov_Pre		
Trust in FDA[3,6]	In your opinion, does the Food and Drug Administration, or FDA, give trustworthy information to the public?	5 = Definitely yes<br>4 = Probably yes<br>3 = Neither yes or no<br>2 = Probably no<br>1 = Definitely no	FDA_Pre		
Trust in CDC[3,6]	In your opinion, does the Centers for Disease Control and Prevention, or CDC, give trustworthy information to the public?	5 = Definitely yes<br>4 = Probably yes<br>3 = Neither yes or no<br>2 = Probably no<br>1 = Definitely no	CDC_Pre		
Page Break					
Nicotine Dependence[7]	How soon after you wake up do you smoke your first cigarette?	3 = Within 5 minutes<br>2 = 6 – 30 minutes	FTND1_Pre	FTND1_Post1	FTND1_Post2

Construct (Source)	Question Text	Response Option	Pretest Var Name	Post 1 Var Name	Post 2 Var Name
		1 = 31-60 minutes<br>0 = After 60 minutes			
	Do you find it difficult to refrain from smoking in places where it is forbidden e.g. in church, at the library, in cinema, etc.?	1 = Yes<br>0 = No	FTND2_Pre	FTND2_Post1	FTND2_Post2
	Which cigarette would you hate most to give up?	1 = The first one in the morning<br>0 = All others	FTND3_Pre	FTND3_Post1	FTND3_Post2
Page Break					
Nicotine Dependence[7]	How many cigarettes per day do you smoke?	0 = 10 or less<br>1 = 11-20<br>2 = 21-30<br>3 = 31 or more	FTND4_Pre	FTND4_Post1	FTND4_Post2
	Do you smoke more frequently during the first hours after waking than during the rest of the day?	1 = Yes<br>0 = No	FTND5_Pre	FTND5_Post1	FTND5_Post2
	Do you smoke if you are so ill that you are in bed most of the day?	1 = Yes<br>0 = No	FTND6_Pre	FTND6_Post1	FTND6_Post2
Page Break					
Previous Quit Attempts[8]	How many times during the past 12 months have you stopped smoking for 1 day or longer because you were trying to quit smoking?	Dropdown (0-365)	PQA_Pre		
Quit Attempts[8]	Since you started this study, how many times have you stopped smoking for 1 day or longer because you were trying to quit smoking?			QuitAtt_Post1<br>Dropdown 0-16	QuitAtt_Post2<br>Dropdown 0-32
Smoking Status[9]	Do you now smoke cigarettes every day, some days or not at all?	2=Every day<br>1=Some days<br>0=Not at all	Smoke_Stat_Pre	Smoke_stat_post1	Smoke_Stat_post2
Page Break					

Construct (Source)	Question Text	Response Option	Pretest Var Name	Post 1 Var Name	Post 2 Var Name
Quit Line Awareness	Do you know what national phone number you can call for help to quit smoking?	1 = Yes<br>0 = No<br>(If no, skip the next question)		QLAware1	
Page Break					
Quit Line Awareness follow up	What is the national phone number?	Text Box		QLAware2	
Page Break					
Quitline Use	Since you started this study, have you called the Quitline (national phone number for help to quit smoking)?	1 = Yes<br>0 = No		QL_Use_Post1	QL_Use_Post2
Page Break					
Quit Intention[4,10]	How interested are you in quitting smoking in the next month?	4=Very interested<br>3=Somewhat interested<br>2=A little interested<br>1=Not at all interested	QI1_Pre	QI1_Post1	QI1_Post2
	How much do you plan to quit smoking in the next month?	4=Very much<br>3=Somewhat<br>2=A little<br>1=Not at all	QI2_Pre	QI2_Post1	QI2_Post2
	How likely are you to quit smoking in the next month?	4=Very likely<br>3=Somewhat likely<br>2=A little likely<br>1=Not at all likely	QI3_Pre	QI3_Post1	QI3_Post2
PAGE BREAK					
Quitline Intention	If you were to quit smoking in the next month, how likely are you to call the national quitline for help?		QLI_Pre	QLI_Post1	QLI_Post2
Littering Intention[4]	How likely are you to dispose of your cigarette butts by dropping them on the ground in the next month?		LI_Pre	LI_Post1	LI_Post2
Page Break					

Construct (Source)	Question Text	Response Option	Pretest Var Name	Post 1 Var Name	Post 2 Var Name
Self Efficacy[11–13]	If you decided to give up smoking completely in the next month, how sure are you that you would succeed?	5=Extremely<br>4=Very much<br>3=Moderately<br>2=A little<br>1=Not at all	SE_Pre	SE_Post1	SE_Post2
Response Efficacy[11]	How much do you think you would benefit from health and other gains if you were to quit smoking permanently in the next month?		RE_Pre	RE_Post1	RE_Post2
PAGE BREAK					
Positive Thoughts[14,15]	During the past 24 hours, how often did positive thoughts about smoking come to mind (thoughts that just popped into your head, or you caught yourself thinking)?	5=All of the time<br>4=Often<br>3=Sometimes<br>2=Rarely<br>1=Never	Freq_Pos_Pre	Freq_Pos_Post1	Freq_Pos_Post2
PAGE BREAK					
Positive Thoughts[14,15]	[displayed if freq_pos ne never]<br><br>Considering only your positive thoughts about smoking during the past 24 hours, how positive were these thoughts?	1=Not at all Positive<br>2=Slightly Positive<br>3=Moderately Positive<br>4=Very Positive<br>5=Extremely Positive	Degree_Pos_Pre	Degree_Pos_Post1	Degree_Pos_Post2
PAGE BREAK					
Negative Thoughts[14,15]	During the past 24 hours, how often did negative thoughts about smoking come to mind (thoughts that just popped into your head, or you caught yourself thinking)?	5=All of the time<br>4=Often<br>3=Sometimes<br>2=Rarely<br>1=Never	Freq_Neg_Pre	Freq_Neg_Post1	Freq_Neg_Post2
PAGE BREAK					
Negative Thoughts[14,15]	[displayed if freq_neg ne never]<br><br>Considering only your negative thoughts about smoking during the past 24 hours, how negative were these thoughts?	1=Not at all Negative<br>2=Slightly Negative<br>3=Moderately Negative<br>4=Very Negative<br>5=Extremely Negative	Degree_Neg_Pre	Degree_Neg_Post1	Degree_Neg_Post2
Page Break					

Construct (Source)	Question Text	Response Option	Pretest Var Name	Post 1 Var Name	Post 2 Var Name
Current OTP Use[16]	In the past 30 days, which of the following products have you used at least once? (Pick all that apply)	1. Cigars, cigarillos, or little cigars<br>2. Smokeless tobacco, for example chewing tobacco, snuff, dip, or snus<br>3. Electronic cigarettes, e-cigarettes, or other vaping devices<br>4. Water pipes or Hookah<br>5. Clove cigarettes<br>6. Roll your own cigarettes<br>7. None of the above [exclusive]	OTPUse_Pre1, OTPUse_Pre2, OTPUse_Pre3, OTPUse_Pre4, OTPUse_Pre5, OTPUse_Pre6, OTPUse_Pre7		
OTP Use 2[16]	Since you started this research study, which of the following products have you used at least once? (Pick all that apply)	1. Cigars, cigarillos, or little cigars<br>2. Smokeless tobacco, for example chewing tobacco, snuff, dip, or snus<br>3. Electronic cigarettes, e-cigarettes, or other vaping devices<br>4. Water pipes or Hookah<br>5. Clove cigarettes<br>6. Roll your own cigarettes<br>7. None of the above [exclusive]		OTPUse_Post1_1, OTPUse_Post1_2, OTPUse_Post1_3, OTPUse_Post1_4, OTPUse_Post1_5, OTPUse_Post1_6, OTPUse_Post1_7	OTPUse_Post2_1, OTPUse_Post2_2, OTPUse_Post2_3, OTPUse_Post2_4, OTPUse_Post2_5, OTPUse_Post2_6, OTPUse_Post2_7
Page Break					
Knowledge[17, 18]	Does cigarette smoke contain…<br>(Matrix Question)	1 = Yes<br>0 = No<br>2 = Don't know	K_Inst1_Pre	K_Inst1_Post1	K_Inst1_Post2

Construct (Source)	Question Text	Response Option	Pretest Var Name	Post 1 Var Name	Post 2 Var Name
	Arsenic		K_As_Pre	K_As_Post1	K_As_Post2
	Ammonia		K_Am_Pre	K_Am_Post1	K_Am_Post2
	Formaldehyde		K_Form_Pre	K_Form_Post1	K_Form_Post2
	Lead		K_Pb_Pre	K_Pb_Post1	K_Pb_Post2
	Uranium		K_U_Pre	K_U_Post1	K_U_Post2
Page Break					
Knowledge[17, 18]	Does cigarette smoke cause…<br>(Matrix Question)	1 = Yes<br>0 = No<br>2 = Don't know	K_Inst2_Pre	K_Inst2_Post1	K_Inst2_Post2
	Brain Disorders		K_BD_Pre	K_BD_Post1	K_BD_Post2
	Breathing Problems		K_BP_Pre	K_BP_Post1	K_BP_Post2
	Cancer		K_Cancer_Pre	K_Cancer_Post1	K_Cancer_Post2
	Heart Damage		K_HD_Pre	K_HD_Post1	K_HD_Post2
	Kidney Damage		K_KD_Pre	K_KD_Post1	K_KD_Post2
	Lung Tumors		K_LT_Pre	K_LT_Post1	K_LT_Post2
	Throat Cancer		K_TC_Pre	K_TC_Post1	K_TC_Post2
Page Break					
Tripartite Risk[19]	If you continue smoking, how **likely** is it that you…	(question in matrix format)	TR1	TR1	TR1
	will get heart damage at some point in the future?	4=Very Likely<br>3=Likely<br>2=Unlikely<br>1=Very Unlikely	TR1_a_Pre	TR1_a_Post1	TR1_a_Post2
	will get throat cancer at some point in the future?		TR1_b_Pre	TR1_b_Post1	TR1_b_Post2
	will get lung tumors at some point in the future?		TR1_c_Pre	TR1_c_Post1	TR1_c_Post2
	will get kidney damage at some point in the future?		TR1_d_Pre	TR1_d_Post1	TR1_d_Post2
	will get breathing problems at some point in the future?		TR1_e_Pre	TR1_e_Post1	TR1_e_Post2
	will get cancer at some point in the future?		TR1_f_Pre	TR1_f_Post1	TR1_f_Post2

Construct (Source)	Question Text	Response Option	Pretest Var Name	Post 1 Var Name	Post 2 Var Name
	will get a brain disorder at some point in the future?		TR1_g_Pre	TR1_g_Post1	TR1_g_Post2
Page Break					
Tripartite Risk[19]	If you continue smoking, how **fearful** are you…	(question in matrix format)	TR2	TR2	TR2
	of getting heart damage in the future?	4=Very fearful<br>3=Fearful<br>2=A little fearful<br>1=Not at all fearful	TR2_a_Pre	TR2_a_Post1	TR2_a_Post2
	of getting throat cancer in the future?		TR2_b_Pre	TR2_b_Post1	TR2_b_Post2
	of getting lung tumors in the future?		TR2_c_Pre	TR2_c_Post1	TR2_c_Post2
	of getting kidney damage in the future?		TR2_d_Pre	TR2_d_Post1	TR2_d_Post2
	of breathing problems damage in the future?		TR2_e_Pre	TR2_e_Post1	TR2_e_Post2
	of getting cancer in the future?		TR2_f_Pre	TR2_f_Post1	TR2_f_Post2
	of getting a brain disorder in the future?		TR2_g_Pre	TR2_g_Post1	TR2_g_Post2
Page Break					
Tripartite Risk[19]	How much do you agree or disagree with the following statement: If I continue to smoke, I feel very vulnerable to…	(question in matrix format)	TR3	TR3	TR3
	heart damage	4=Strongly Agree<br>3=Agree<br>2=Disagree<br>1=Strongly Disagree	TR3_a_Pre	TR3_a_Post1	TR3_a_Post2
	throat cancer		TR3_b_Pre	TR3_b_Post1	TR3_b_Post2
	lung tumors		TR3_c_Pre	TR3_c_Post1	TR3_c_Post2
	kidney damage		TR3_d_Pre	TR3_d_Post1	TR3_d_Post2
	breathing problems		TR3_e_Pre	TR3_e_Post1	TR3_e_Post2
	cancer		TR3_f_Pre	TR3_f_Post1	TR3_f_Post2
	brain disorders		TR3_g_Pre	TR3_g_Post1	TR3_g_Post2
Page Break					
FDA Information[3,6]	The Food and Drug Administration, or FDA, is the agency that ensures that food and drugs sold in this country are safe.			FDA_Info_Post1	

Construct (Source)	Question Text	Response Option	Pretest Var Name	Post 1 Var Name	Post 2 Var Name
	The FDA now regulates cigarettes and some other tobacco products. The next series of questions is about your opinions toward FDA in this role.				
FDA Credibility[3,6]	Do you trust the FDA to inform the public about the risks of tobacco products?	5 = Definitely yes<br>4 = Probably yes<br>3 = Neither yes or no<br>2 = Probably no<br>1 = Definitely no		Cred1_Post1	
	Is the FDA honest about the risks of tobacco products?			Cred2_Post1	
	Do you believe what the FDA says about the risks of tobacco products?			Cred3_Post1	
	Is the FDA an expert on regulating tobacco products?			Cred4_Post1	
	Is the FDA capable of doing a good job regulating tobacco products?			Cred5_Post1	
	Can the FDA effectively regulate tobacco products?			Cred6_Post1	
	Is the FDA committed to protecting the public from possible risks of tobacco products?			Cred7_Post1	
	Do you believe that if the FDA knew that certain tobacco products are less harmful than thought, they would tell the public?			Cred8_Post1	
Page Break					
Mental Health[20]	During the past 30 days, about how often did you feel…	(question in matrix format)	MH1	MH1	MH1
	nervous?	4=All of the time<br>3=Most of the time<br>2=Some of the time<br>1=A little of the time<br>0=None of the time	MH1_a_Pre	MH1_a_Post1	MH1_a_Post2
	hopeless?		MH1_b_Pre	MH1_b_Post1	MH1_b_Post2
	restless or fidgety?		MH1_c_Pre	MH1_c_Post1	MH1_c_Post2
	so depressed that nothing could cheer you up?		MH1_d_Pre	MH1_d_Post1	MH1_d_Post2

Construct (Source)	Question Text	Response Option	Pretest Var Name	Post 1 Var Name	Post 2 Var Name
	that everything was an effort?		MH1_e_Pre	MH1_e_Post1	MH1_e_Post2
	worthless?		MH1_f_Pre	MH1_f_Post1	MH1_f_Post2
Page Break					
Instruction	Please select the bubble to answer the questions about the messages that you saw during the study:			AE_Inst_Post1	AE_Inst_Post2
Action expectancies [21]	After seeing the messages, how likely are you to seek information about chemicals in cigarettes?	4=Very Likely<br>3=Likely<br>2=Unlikely<br>1=Very Unlikely		AE1_Post1	AE1_Post2
	After seeing the messages, how likely are you to seek help to quit smoking?	4=Very Likely<br>3=Likely<br>2=Unlikely<br>1=Very Unlikely		AE2_Post1	AE2_Post2
Page Break					
Instruction	Please select the bubble to show how much you agree or disagree with the statements below about the messages that you saw during the study:			React	
Reactance[22]	The messages were trying to manipulate me	5=Strongly agree<br>4=Somewhat agree<br>3=Neither agree nor disagree<br>2=Somewhat disagree<br>1=Strongly disagree		React_1_Post1	
	The messages made me feel aggravated			React_2_Post1	
	The health effects on the messages were overblown			React_3_Post1	
Page Break					
Conversations[23,24]	During the study, did you talk to others about the messages you received about cigarettes?	1=yes<br>0=no		Convo1_Post1	

Construct (Source)	Question Text	Response Option	Pretest Var Name	Post 1 Var Name	Post 2 Var Name
Page Break					
Conversations[24]	[only display if convo1_Post1 = 1]<br>When you talked about the messages, would you say your conversations were mostly…	1=Negative about smoking cigarettes<br>2=Neutral about smoking cigarettes<br>3=Positive about smoking cigarettes		Convo2_Post1	
Page Break					
Behavior Instructions	Please think about yesterday		Behav_Inst	Behav_Inst	Behav_Inst
	What time did you wake up yesterday?	Time	Time#1_Wake_Hour<br>Time#1_Sleep_Hour<br>Time#2_Wake_Min<br>Time#2_Sleep_Min<br>Time#3_Wake_AMPM<br>Time#3_Sleep_AMPM		
	What time did you go to sleep last night?	Time			
Behavior[4]	How many times yesterday did you dispose of your cigarette butts by dropping them on the ground?	Dropdown list 0-100, 101=refused	Lit1	Lit1	Lit1
	How many times yesterday did you dispose of your cigarette butts by throwing them or dropping them out of the car window?		Lit2	Lit2	Lit2
	How many times yesterday did you stop yourself from having a cigarette because you wanted to smoke less?		Forgo	Forgo	Forgo
	How many times yesterday did you butt out a cigarette before you finished because you wanted to smoke less?		Butt	Butt	Butt
PAGE BREAK					
Cigarettes Smoked[7]	Yesterday, from the time you woke up until noon, how many cigarettes did you smoke?	Dropdown list 0-100, 101=refused	Cigs_Morn	Cigs_Morn	Cigs_Morn

Construct (Source)	Question Text	Response Option	Pretest Var Name	Post 1 Var Name	Post 2 Var Name
	Yesterday, from noon until you went to sleep, how many cigarettes did you smoke?		Cigs_Afternoon	Cigs_Afternoon	Cigs_Afternoon
PAGE BREAK					
Thinking Instructions[4]	Overall yesterday, how much did you…		Think	Think	Think
Thinking About Chemicals[4]	Think about the chemicals in the smoke from your cigarettes?	5=All of the time<br>4=Often<br>3=Sometimes<br>2=Rarely<br>1=Never	Think_Chem	Think_Chem	Think_Chem
Thinking About Risks	Think about the harm your smoking might be doing to you?		Think_Risk	Think_Risk	Think_Risk
Thinking About Littering	Think about discarding cigarette butts properly?		Think_Lit	Think_Lit	Think_Lit
Thinking about quitting	Think about quitting smoking?		Think_Quit	Think_Quit	Think_Quit
PAGE BREAK					
StudyInfo1	The next few pages give you some more information for what to expect during the study.		StudyInfo1		
PAGE BREAK			PAGE BREAK		
StudyInfo2	Starting tomorrow, you will receive an email at 7am every morning for 15 days from 'admin@SurveySignal.com', with a link to a short survey. You have until 11am to open the link and answer the survey, or you will have to wait until the next one is sent.<br>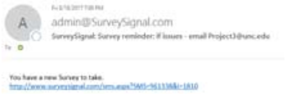		StudyInfo2		
PAGE BREAK			PAGE BREAK		

Construct (Source)	Question Text	Response Option	Pretest Var Name	Post 1 Var Name	Post 2 Var Name
StudyInfo4	After 15 days (16 days from now), you will receive another email at 7am with a link to a 20-minute survey. You will have 24 hours to open the link and answer the survey.<br><br>Finally, you will receive another email 32 days from now with a link to a 20-minute survey. You will have 24 hours to open the link and answer the survey.		StudyInfo4		
PAGE BREAK			PAGE BREAK		
StudyInfo5	You will be paid for the study based on how many surveys you complete with a check that will arrive in the mail after you complete the study.<br><br>You will receive $20 for completing this survey.<br><br>For the daily morning surveys, you will be paid $4 for each survey that you complete.<br><br>For the 20-minute surveys at the end of the study, you will receive $20 each if you complete them.<br><br>When data collection for the full study is complete, we will have a drawing for three Amazon gift cards, with a $200 value. Entries into the drawing will be based on completion of the morning surveys.<br>**Bonus system for Morning Survey Completion:**<br>• Bronze: Total of $10 bonus for at least 9 morning surveys. 1 entry into Amazon gift card drawing.		StudyInfo5		

Construct (Source)	Question Text	Response Option	Pretest Var Name	Post 1 Var Name	Post 2 Var Name
	• Silver: Total of $20 bonus for at least for at least 12 morning surveys. 2 entries into Amazon gift card drawing.<br>• Gold: Total of $30 bonus for completing all 15 morning surveys. 3 entries into Amazon gift card drawing.<br><br>If you complete all the surveys for this study, your check amount will be $150.				
PAGE BREAK					
Quality Assurance	When you took the surveys, ere you able to see each message?	0=No<br>1=Yes		QA1	
PAGE BREAK					
Quality Assurance	[IF QA1 = No] Why weren't you able to see each message, when you took the surveys?	[text box]		QA1 _comment	
PAGE BREAK					
Quality Assurance	Were you able to read the text on each of the messages?	0=No<br>1=Yes		QA2	
PAGE BREAK					
Quality Assurance	[IF QA2 = No] Why weren't you able to read the text on each of the messages?	[text box]		QA2_comment	
PAGE BREAK					
Quality Assurance	Did you understand all of the questions?	0=No<br>1=Yes		QA3	
PAGE BREAK					
Quality Assurance	[IF QA3 = No] Which questions did you have trouble understanding and why?	[text box]		QA3_comment	
PAGE BREAK					
Quality Assurance	Would you do this study again?	0=No<br>1=Yes		QA4	

Construct (Source)	Question Text	Response Option	Pretest Var Name	Post 1 Var Name	Post 2 Var Name
Quality Assurance	Would you recommend this study to a friend?	0=No<br>1=Yes		QA5	
Quality Assurance	Did anything annoy you, if so, what?	[text box]		QA6	
Quality Assurance	Is there anything that you would like to share about your experience in the study?	[text box]		QA7	
Quality Assurance	Did you have any difficulty remembering how many cigarettes you smoked? If yes, please explain.	[text box]		QA8	
Quality Assurance	Would you have preferred to receive the surveys for this study via text message?	0=No<br>1=Yes		QA9	
PAGE BREAK					
Instructions for Enrolling in Survey Signal	On the next page, you will be redirected to a new link (SurveySignal). **It is very important that you fill out the information on that page** (including name, email address, etc.). If you do not fill out that information, you will NOT be enrolled in the study. If you have any questions, email Project3@unc.edu.<br><br>Once you fill in your information, SurveySignal will send you an email to verify your email address. Please make sure that you verify your email, so that you will be enrolled in the study.<br><br>**Note:** If you are using a Mac, you will need to open the link in Chrome or Firefox.		SurveySignal_Inst		
End of Survey for anyone that is no longer a	Unfortunately you are no longer eligible for our study. We will send you a check for $20 to compensate you for your time completing this survey, but you will not be able to complete the rest of the study.				

Construct (Source)	Question Text	Response Option	Pretest Var Name	Post 1 Var Name	Post 2 Var Name
smoker (smoke_stat_ pre =0)	Thank you for taking the time to complete this survey!				

Morning Questionnaire (to be answered between 7am – 11am every morning)

Construct (Source)	Question Text	Response Option	Var Name
Study ID	[Auto-recorded]	numerical	
Behavior Instructions	Please think about yesterday		Behav_Inst
	What time did you wake up yesterday?	Time	Time#1_Wake_Hour<br>Time#1_Sleep_Hour<br>Time#2_Wake_Min<br>Time#2_Sleep_Min<br>Time#3_Wake_AMPM<br>Time#3_Sleep_AMPM
	What time did you go to sleep last night?	Time	
Behavior[4]	How many times yesterday did you dispose of your cigarette butts by dropping them on the ground?	Dropdown list 0-100, 101=refused	Lit1
	How many times yesterday did you dispose of your cigarette butts by throwing them or dropping them out of the car window?		Lit2
	How many times yesterday did you stop yourself from having a cigarette because you wanted to smoke less?		Forgo
	How many times yesterday did you butt out a cigarette before you finished because you wanted to smoke less?		Butt
PAGE BREAK			
Cigarettes Smoked[7]	Yesterday, from the time you woke up until noon, how many cigarettes did you smoke?	Dropdown list 0-100, 101=refused	Cigs_Morn
	Yesterday, from noon until you went to sleep, how many cigarettes did you smoke?		Cigs_Afternoon
PAGE BREAK			
Thinking Instructions[4]	Overall yesterday, how much did you…		Think
Thinking About Chemicals[4]	Think about the chemicals in the smoke from your cigarettes?	5=All of the time<br>4=Often<br>3=Sometimes<br>2=Rarely<br>1=Never	Think_Chem
Thinking About Risks	Think about the harm your smoking might be doing to you?		Think_Risk
Thinking About Littering	Think about discarding cigarette butts properly?		Think_Lit
Thinking about quitting	Think about quitting smoking?		Think_Quit

Construct (Source)	Question Text	Response Option	Var Name
PAGE BREAK			
Instruction	Please view the message on the next screen. After 10 seconds, you will be able to click the ‘>>’ to move forward to the next page.<br><br>[Messages should appear at the top of the screen for all subsequent questions]		Morn_Inst1
Page Break			
Message	Condition 1 or 2(Arsenic, Formaldehyde, Uranium, Ammonia, Lead) Or Control Messages [This should appear on the screen for 10 seconds before participants are able to move on to questions on the next page]		Message
Page Break			
Affect Instruction	How much did the **message** make you feel…		Affect_Inst
Affect[25–27]	Anxious?	5=Extremely<br>4=Very<br>3=Somewhat<br>2=A little<br>1=Not at all	Affect_1
	Sad?		Affect_2
	Scared?		Affect_3
	Guilty?		Affect_4
	Disgusted?		Affect_5
Page Break			
Credibility[28]	Please indicate how much you think the message is…		Cred_Inst
	Accurate	7 = Very accurate<br>1 = Not very accurate	Cred_1
	Authentic	7 = Very authentic<br>1 = Not very authentic	Cred_2
	Believable	7 = Very believable<br>1 = Not very believable	Cred_3
Page Break			

Construct (Source)	Question Text	Response Option	Var Name
Instruction	Please select the bubble to show how much you agree or disagree with the statements below:		PE_Inst
Perceived Effectiveness[29]	The message makes me concerned about the health effects of smoking.	5=Strongly agree<br>4=Somewhat agree<br>3=Neither agree nor disagree<br>2=Somewhat disagree<br>1=Strongly disagree	PE_1
	The message makes smoking seem unpleasant to me.		PE_2
	The message discourages me from wanting to smoke.		PE_3
PAGE BREAK			
	How many cigarettes do you think you will smoke today?	Dropdown list 0-100, 101=refused	Cigs_Predict
PAGE BREAK			
End of Survey	You're on day X of the 15 days of morning surveys. You've completed X morning survey(s) so far. We appreciate your response(s). Thank you!		

References for Measures

1. The GenIUSS Group. *Best Practices for Asking Questions to Idenfity Transgender and Other Gender Minority Respondents on Population-Based Surveys*.; 2014. https://williamsinstitute.law.ucla.edu/wp-content/uploads/geniuss-report-sep-2014.pdf. Accessed June 22, 2018.
2. Sexual Minority Assessment Research Team (SMART). *Best Practices for Asking Questions about Sexual Orientation on Surveys*.; 2009. https://williamsinstitute.law.ucla.edu/wp-content/uploads/SMART-FINAL-Nov-2009.pdf. Accessed June 22, 2018.
3. Boynton MH, Agans RP, Bowling JM, et al. Understanding how perceptions of tobacco constituents and the FDA relate to effective and credible tobacco risk messaging: A national phone survey of U.S. adults, 2014–2015. *BMC Public Health*. 2016;16(1):516. doi:10.1186/s12889-016-3151-5
4. Brewer NT, Jeong M, Mendel JR, et al. Cigarette pack messages about toxic chemicals: a randomised clinical trial. *Tob Control*. April 2018:tobaccocontrol-2017-054112. doi:10.1136/tobaccocontrol-2017-054112
5. Department of Health and Human Services. *2017 Annual Update of the HHS Poverty Guidelines*. Vol 82.; 2017. https://www.gpo.gov/fdsys/pkg/FR-2017-01-31/pdf/2017-02076.pdf. Accessed June 22, 2018.
6. Schmidt AM, Jarman KL, Ranney LM, et al. Public Knowledge and Credibility Perceptions of the FDA as a Tobacco Regulator. *Nicotine Tob Res*. September 2017. doi:10.1093/ntr/ntx215
7. Heatherton TF, Kozlowski LT, Frecker RC, Fagerström KO. The Fagerström Test for Nicotine Dependence: a revision of the Fagerström Tolerance Questionnaire. *Br J Addict*. 1991;86(9):1119-1127. http://www.ncbi.nlm.nih.gov/pubmed/1932883. Accessed February 25, 2018.
8. Fagan P, Augustson E, Backinger CL, et al. Quit attempts and intention to quit cigarette smoking among young adults in the United States. *Am J Public Health*. 2007;97(8):1412-1420. doi:10.2105/AJPH.2006.103697
9. Centers for Disease Control and Prevention. *2016 Behavioral Risk Factor Surveillance System Questionnaire*.; 2016.
10. Klein WMP, Zajac LE, Monin MM. Worry as a Moderator of the Association Between Risk Perceptions and Quitting Intentions in Young Adult and Adult Smokers. *Ann Behav Med*. 2009;38(3):256-261. doi:10.1007/s12160-009-9143-2
11. Thrasher JF, Swayampakala K, Borland R, et al. Influences of Self-Efficacy, Response Efficacy, and Reactance on Responses to Cigarette Health Warnings: A Longitudinal Study of Adult Smokers in Australia and Canada. *Health Commun*. 2016;31(12):1517-1526. doi:10.1080/10410236.2015.1089456
12. Thrasher JF, Swayampakala K, Cummings KM, et al. Cigarette package inserts can promote efficacy beliefs and sustained smoking cessation attempts: A longitudinal assessment of an innovative policy in Canada. *Prev Med (Baltim)*. 2016;88:59-65. doi:10.1016/j.ypmed.2016.03.006
13. IARC HANDBOOKS OF CANCER PREVENTION. *Int Agency Res Cancer World Heal Organ*. 12. http://www.iarc.fr/en/publications/pdfs-online/prev/handbook12/Tobacco_vol12_cover.pdf. Accessed June 21, 2018.
14. Rice EL, Fredrickson BL. Do positive spontaneous thoughts function as incentive salience? *Emotion*. 2017;17(5):840-855. doi:10.1037/emo0000284
15. Orbell S, Hagger M. Temporal framing and the decision to take part in type 2 diabetes screening: Effects of individual differences in consideration of future consequences on persuasion. *Heal Psychol*. 2006;25(4):537-548. doi:10.1037/0278-6133.25.4.537
16. Regan AK, Promoff G, Dube SR, Arrazola R. Electronic nicotine delivery systems: adult use and awareness of the "e-cigarette" in the USA. *Tob Control*. 2013;22(1):19-23. doi:10.1136/tobaccocontrol-2011-050044

17. Lazard AJ, Byron MJ, Vu H, Peters E, Schmidt A, Brewer NT. Website Designs for Communicating About Chemicals in Cigarette Smoke. *Health Commun*. 2019;34(3):333-342. doi:10.1080/10410236.2017.1407276

18. Rohde JA, Noar SM, Horvitz C, Lazard AJ, Ross JC, Sutfin EL. The role of knowledge and risk beliefs in adolescent E-cigarette use: A pilot study. *Int J Environ Res Public Health*. 2018;15(4). doi:10.3390/ijerph15040830

19. Ferrer RA, Klein WMP, Persoskie A, Avishai-Yitshak A, Sheeran P. The tripartite model of risk perception (TRIRISK): distinguishing deliberative, affective, and experiential components of perceived risk. *Ann Behav Med*. 2016;50(5):653-663. doi:10.1007/s12160-016-9790-z

20. Kessler RC, Barker PR, Colpe LJ, et al. Screening for serious mental illness in the general population. *Arch Gen Psychiatry*. 2003;60(2):184-189. doi:10.1001/archpsyc.60.2.184

21. Osman A, Kowitt SD, Sheeran P, Jarman KL, Ranney LM, Goldstein AO. Information to Improve Public Perceptions of the Food and Drug Administration (FDA's) Tobacco Regulatory Role. *Int J Environ Res Public Health*. 2018;15(4). doi:10.3390/ijerph15040753

22. Hall MG, Sheeran P, Noar SM, et al. Negative affect, message reactance and perceived risk: how do pictorial cigarette pack warnings change quit intentions? *Tob Control*. December 2017:tobaccocontrol-2017-053972. doi:10.1136/tobaccocontrol-2017-053972

23. Morgan JC, Southwell BG, Noar SM, Ribisl KM, Golden SD, Brewer NT. Frequency and Content of Conversations About Pictorial Warnings on Cigarette Packs. *Nicotine Tob Res*. 2018;20(7):882-887. https://pubmed.ncbi.nlm.nih.gov/29059415/. Accessed July 13, 2020.

24. Noar SM, Rohde JA, Horvitz C, Lazard AJ, Cornacchione Ross J, Sutfin EL. Adolescents' receptivity to E-cigarette harms messages delivered using text messaging. *Addict Behav*. June 2018. doi:10.1016/j.addbeh.2018.05.025

25. Nonnemaker J, Farrelly M, Kamyab K, Busey A, Mann N. *Experimental Study of Graphic Cigarette Warning Labels: Final Results Report*.; 2010. http://www.tobaccolabels.ca/wp/wp-content/uploads/2013/12/USA-2010-Experimental-Study-of-Graphic-Cigarette-Warning-Labels-Final-Results-Report-FDA.pdf. Accessed July 11, 2018.

26. Keller PA, Block LG. Increasing the persuasiveness of fear appeals: The effect of arousal and elaboration. *J Consum Res*. 1996;22(4):448-459. doi:10.1086/209461

27. Watson D, Clark L, Tellegen A. Development and validation of brief measures of positive and negative affect: the PANAS scales - PubMed. *J Pers Soc Psychol*. 1988;54(6):1063-1070. https://pubmed.ncbi.nlm.nih.gov/3397865/. Accessed August 7, 2020.

28. Appelman A, Sundar SS. Measuring Message Credibility. *Journal Mass Commun Q*. 2016;93(1):59-79. doi:10.1177/1077699015606057

29. Baig SA, Noar SM, Gottfredson NC, Boynton MH, Ribisl KM, Brewer NT. UNC Perceived Message Effectiveness: Validation of a Brief Scale. *Manuscr Submitt Publ*. 2018.

Appendix B: Trial Consent Form

University of North Carolina at Chapel Hill
Consent to Participate in a Research Study
Adult Participants

Consent Form Version Date: V6, December 18, 2017
IRB Study # 17-0610
Title of Study: Enhancing Source Credibility in Tobacco Regulatory Communications - Aim 3 RCT
Principal Investigator: Adam Goldstein
Principal Investigator Department: Family Medicine
Principal Investigator Phone number: (919) 966-4090
Principal Investigator Email Address: aog@med.unc.edu
Co-Investigators: Leah Ranney, Seth Noar, Paschal Sheeran
Funding Source and/or Sponsor: National Institutes of Health (NIH)
Study Coordinator Contact: Kristen Jarman
Study Contact Telephone Number: (919) 966-3016
Study Contact Email: project3@unc.edu

What are some general things you should know about research studies?
You are being asked to take part in a research study. To join the study is voluntary.
You may choose not to participate, or you may withdraw your consent to be in the study, for any reason, without penalty.

Research studies are designed to obtain new knowledge. This new information may help people in the future. You may not receive any direct benefit from being in the research study. There also may be risks to being in research studies.

Details about this study are discussed below. It is important that you understand this information so that you can make an informed choice about being in this research study.

You will be given a copy of this consent form. You should ask the researchers named above, or staff members who may assist them, any questions you have about this study at any time.

What is the purpose of this study?
The purpose of this research study is to learn how people respond to messages about cigarette use. We will be asking participants to complete several surveys over 33 days, to get unique data about how people respond to messages about cigarettes.

Who should take part in the study?
Only participants that have been asked to enroll in the study are eligible. If you did not receive an email invitation to take part in the study, and you enroll anyway, you will be removed from the study. Additionally, each person can only enroll in the study once.

How many people will take part in this study?
There will be approximately 1000 people in this research study.

How long will your part in this study last?

Your part in the study will be to answer surveys delivered to your email over 33 days.

What will happen if you take part in the study?

You will receive emails during the study period to invite you to complete surveys. The chart below describes which days you will receive surveys and how long they will be.

Day 1 - 20 minute survey → Day 2 - Day 16 • Morning - 5 min survey → Day 17 - 20 minute survey → Day 33 - 20 minute survey

What are the possible benefits from being in this study?

By taking part in this study, participants may increase their knowledge of the health risks associated with tobacco use. All participants may experience personal satisfaction of knowing they have contributed to a research project aimed at understanding tobacco risk communications.

What are the possible risks or discomforts involved from being in this study?

Sometimes things happen to people in research studies that may make them feel bad. These are called "risks." Taking part in this research study may involve telling us about your opinions or behaviors that you consider to be private or that cause you embarrassment. We will take steps to keep your information safe and private.

There may be uncommon or previously unknown risks. You should report any problems to the researcher.

What if we learn about new findings or information during the study?

You will be given any new information gained during the course of the study that might affect your willingness to continue your participation.

How will information about you be protected?

Participants will not be identified in any report or publication about this study. Although every effort will be made to keep research records private, there may be times when federal or state law requires the disclosure of such records, including personal information. This is very unlikely, but if disclosure is ever required, UNC-Chapel Hill will take steps allowable by law to protect the privacy of personal information. In some cases, your information in this research study could be reviewed by representatives of the University, research sponsors, or government agencies (for example, the FDA) for purposes such as quality control or safety.

What if you want to stop before your part in the study is complete?

You can withdraw from this study at any time, without penalty. The investigators also have the right to stop your participation at any time. This could be because you have had an unexpected reaction, or have failed to follow instructions, or because the entire study has been stopped.

Will you receive anything for being in this study?

You will be receiving a check for up to $150 for taking part in this study. The amount of the check is based on the number of study surveys that you complete.

At the beginning (day 1), middle (day 17) and end (day 33) of this study there are 3 surveys that will take about 20 minutes to complete. You will be paid $20 for each of these surveys that you complete, for up to $60.

For 15 days (from day 2 through day 16) during this study, you will receive a survey in the morning that will last about 5 minutes each to complete, you must complete this survey between 7am and 11am. You will be paid $4 for each morning survey, but you can only take one morning survey each day. You can also earn bonuses for completing these survey.

When data collection for the full study is complete, we will have a drawing for three additional Amazon gift cards, with a $200 value. Entries into the drawing will be based on completion of the morning surveys.

Bonus system for Survey Completion:

- Bronze: Total of $10 bonus for at least 9 morning surveys. 1 entry into Amazon gift card drawing.
- Silver: Total of $20 bonus for at least 12 morning surveys. 2 entries into Amazon gift card drawing.
- Gold: Total of $30 for completing all 15 morning surveys. 3 entries into Amazon gift card drawing.

For Example: If Amy completes 2 of the 3 surveys that take 20 minutes, and 13 morning surveys, then she would get:

- **2 x $20 for 20 minute surveys = $40**
- **13 x $4 for morning surveys = $52**
- **Silver level for survey completion = $20**
- **Amy's Check Amount = $112**

Within 4-6 weeks of completing the study, you will be mailed the check for surveys that you have completed.

If you win the drawing for the Amazon gift card, we will email you the gift card within 7 days of the drawing.

Will it cost you anything to be in this study?
If you enroll in this study, you will have costs related to completing the surveys on the internet. If you use your phone to complete the surveys, this may cost you a small amount of data from your mobile phone plan which will not be covered by the study.

Who is sponsoring this study?
This research is funded by the National Cancer Institute. This means that the research team is being paid by the sponsor for doing the study. The researchers do not, however, have a direct financial interest with the sponsor or in the final results of the study.

What if you have questions about this study?
You have the right to ask, and have answered, any questions you may have about this research. If you have questions about the study (including payments), complaints, concerns, or if a research-related injury occurs, you should contact the researchers listed on the first page of this form.

What if you have questions about your rights as a research participant?
All research on human volunteers is reviewed by a committee that works to protect your rights and welfare. If you have questions or concerns about your rights as a research subject, or if you would like to obtain information or offer input, you may contact the Institutional Review Board at 919-966-3113 or by email to IRB_subjects@unc.edu.

Participant's Agreement:

I have read the information provided above. I have asked all the questions I have at this time. I voluntarily agree to participate in this research study.

[Check Box]
