# Supplementary material for: Effect of Cigarette Constituent Messages With Engagement Text on Intention to Quit Smoking Among Adults Who Smoke Cigarettes: A Randomized Clinical Trial
Source: JAMA Netw Open. 2021 Feb 24;4(2):e210045. doi: 10.1001/jamanetworkopen.2021.0045 (PMC7905497; doi:10.1001/jamanetworkopen.2021.0045)
Supplement: Supplement 2. — eAppendix. Details Regarding Statistical Analysis eFigure 1. Messages by Condition eFigure 2. Quit Intentions at Pretest, Day 16, and Day 32 eTable 1. Quit Intentions at Day 16 and Day 32, Without the Dose Variable eTable 2. Secondary Behavioral Outcomes at Day 32, Without the Dose Variable eTable 3. Quit Attempts at Day 32, Without the Dose Variable eTable 4. Summary Data on Quit Intentions, Secondary Behavioral Outcomes, and Quit Attempts at Pretest, Day 16, and Day 32 eTable 5. Quit Attempts at Day 32 eReferences. [file jamanetwopen-e210045-s002.pdf]

Supplemental Online Content

Goldstein AO, Jarman KL, Kowitt SD, et al. Effect of cigarette constituent messages with engagement text on intention to quit smoking among adults who smoke cigarettes: a randomized clinical trial. *JAMA Netw Open*. 2021;4(2):e210045. doi:10.1001/jamanetworkopen.2021.0045

eAppendix. Details Regarding Statistical Analysis

eFigure 1. Messages by Condition

eFigure 2. Quit Intentions at Pretest, Day 16, and Day 32

eTable 1. Quit Intentions at Day 16 and Day 32, Without the Dose Variable

eTable 2. Secondary Behavioral Outcomes at Day 32, Without the Dose Variable

eTable 3. Quit Attempts at Day 32, Without the Dose Variable

eTable 4. Summary Data on Quit Intentions, Secondary Behavioral Outcomes, and Quit Attempts at Pretest, Day 16, and Day 32

eTable 5. Quit Attempts at Day 32

eReferences.

This supplemental material has been provided by the authors to give readers additional information about their work.

© 2021 Goldstein AO et al. *JAMA Network Open.*

eAppendix. Details Regarding Statistical Analysis

Multiple Imputation Procedures for our Primary Outcome: Prior to conducting the multiple imputation procedure, we examined missing data patterns. First, we assessed the proportion of missing values among our primary and secondary outcomes of interest. Based on our missing data pattern analysis, we ruled out a missing completely at random pattern, and assumed that data are missing at random, and analyzed the data based on this assumption. The missing at random assumption is more realistic than missing completely at random for most studies.[1] Multiple imputation procedures assuming missing at random produce an accurate set of estimates.[2,3] We used a Markov Chain Monte Carlo method to impute all the missing values.

Power analysis: The primary outcome of the study was quit intentions at post-test 1 (day 16). Our power analysis determined the sample size necessary to compare quit intentions at post-test 1 to quit intentions at baseline between the 3 treatment groups. In a meta-analysis by Noar et al. 2016,[4] the effect size between pictorial vs. text only warnings on intention to quit smoking was a standardized mean difference (Cohen's *d*) of 0.54. We assumed 1) that the smallest difference in quit intention changes would be between our optimal and suboptimal constituent messages, 2) a continuous measure of quit intention, 3) that adding self-efficacy text would make a smaller difference in quit intention than adding pictorial images (the focus of the Noar review).[4] As a result, we set our effect size at d=0.25. To achieve 80% power between two independent groups in a t-test at α=0.05, group sizes must be 253. More complex power simulations yielded a similar sample size per group, for a total of around 750 participants. To be conservative, we planned to enroll 800 participants.

Imputation Procedure used for our Primary Outcome: We used a Markov Chain Monte Carlo method to impute all the missing values, consisting of the following three steps:

Step 1: Imputation Phase. We imputed the missing data with estimated values to create a complete dataset. We employed a Markov Chain Monte Carlo method, which is the most widely used parametric method and assumes that all the variables in the imputation model follow a joint multivariate normal distribution. We repeated this imputation process 5 times.

Step 2: Analysis Phase. We analyzed each of the imputed datasets individually by using general linear modeling.

Step 3: Pooling Phase. We combined all the parameter estimates across all imputation datasets from Step 2 to provide inference that represents the uncertainty about the predictions of the missing values using Rubin's rules. We combined the individual coefficients and standard errors for each of 5 regression models.[5] With 5 imputations, we computed the parameter and its variance from 5 different data sets. Let M_i, and W_i be the parameter estimates and their variance estimates from the ith imputed data set, i=1,2,…,5, respectively. Then we suppose that M_{ave} is the combined parameter estimate, which is the average of the 5 parameters from each model. Suppose W_w is the within-imputation variance, which is the average of the 5 complete-data estimates and W_b is the between-imputation variance. Then the variance estimate associated with the individual coefficient is the total variance, saying it as T. (Rubin, 1987)

Note:

$$M_{ave} = \frac{1}{5}\sum_{i=1}^{5} M_i,$$

$$W_w = \frac{1}{5}\sum_{i=1}^{5} W_i,$$

$$W_b = \frac{1}{4}\sum_{i=1}^{5}(M_i - M_{ave})^2,$$

$$T = W_w + \left(1 + \frac{1}{5}\right) W_b = W_w + \frac{6}{5} W_b$$

© 2021 Goldstein AO et al. *JAMA Network Open.*

eFigure 1. Messages by Condition

<table>
<tr><th>Constituent plus Engagement Message Group</th><th>Constituent Only Message Group</th><th>Control Littering Message Group</th></tr>
<tr>
<td>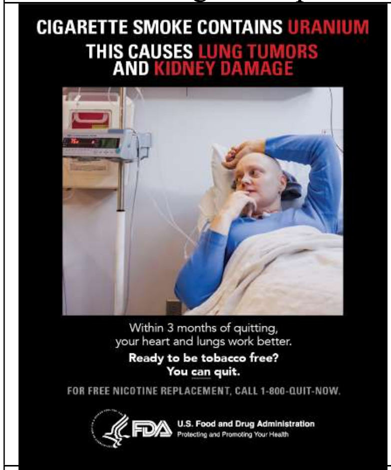
</td>
<td>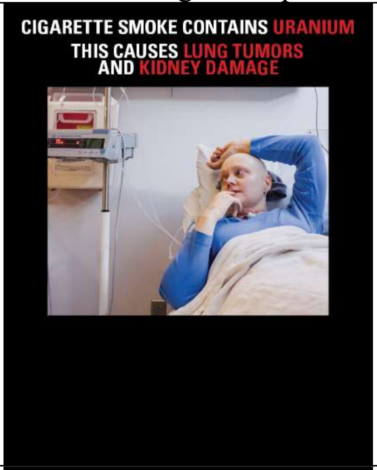
</td>
<td>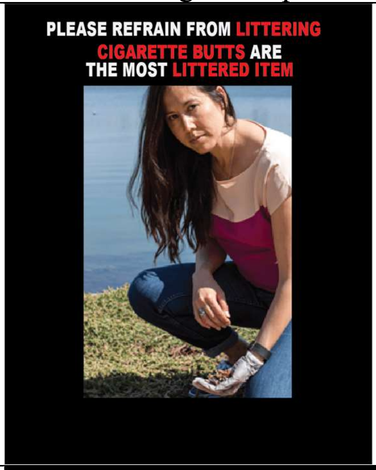
</td>
</tr>
<tr>
<td>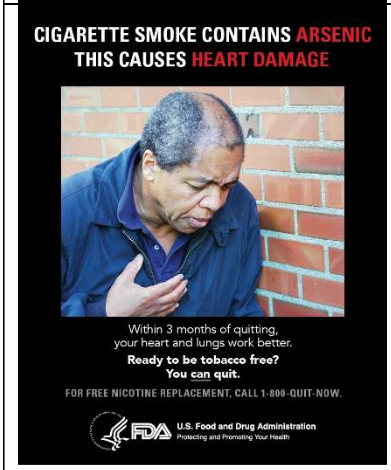
</td>
<td>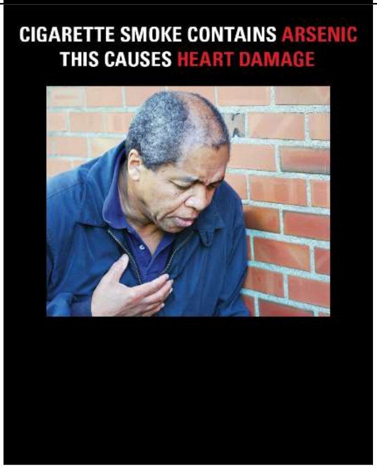
</td>
<td>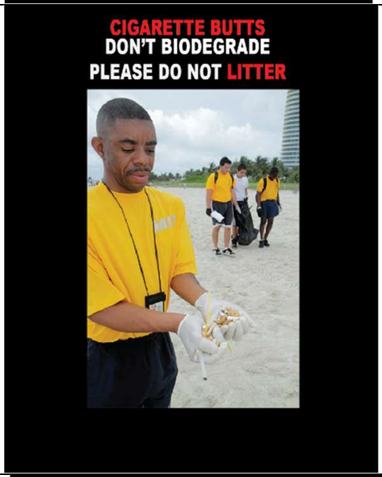
</td>
</tr>
<tr>
<td>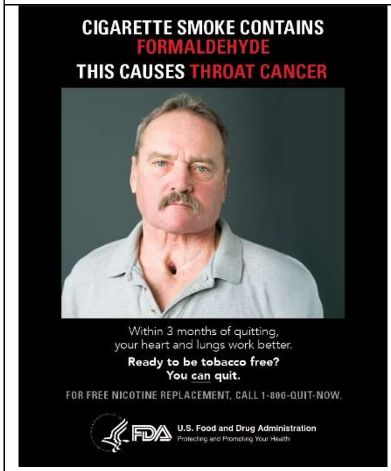
</td>
<td>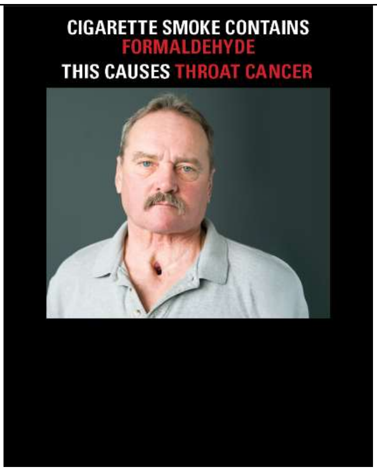
</td>
<td>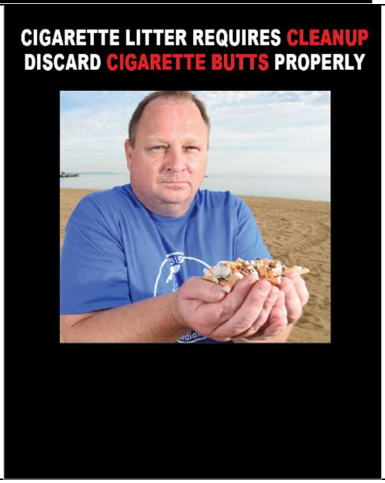
</td>
</tr>
</table>

© 2021 Goldstein AO et al. *JAMA Network Open.*

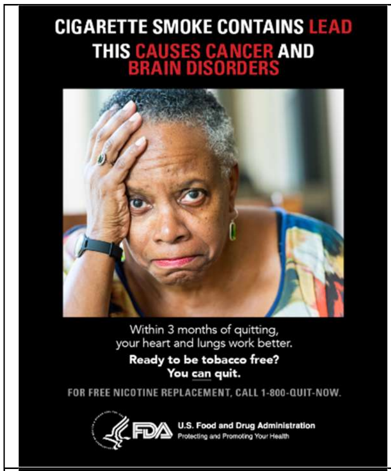

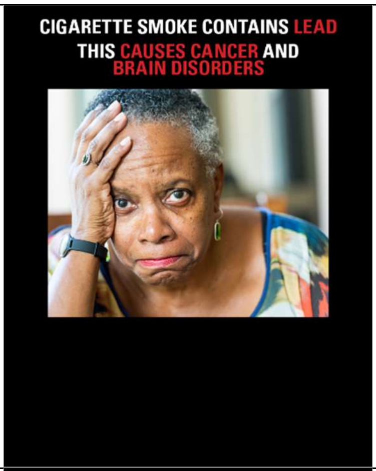

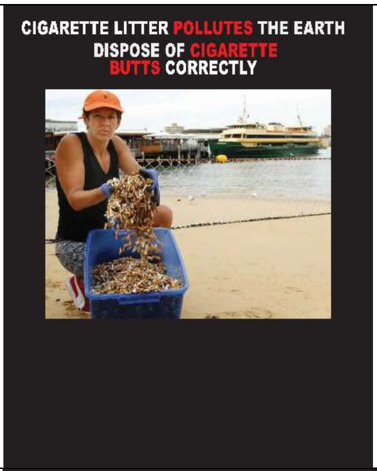

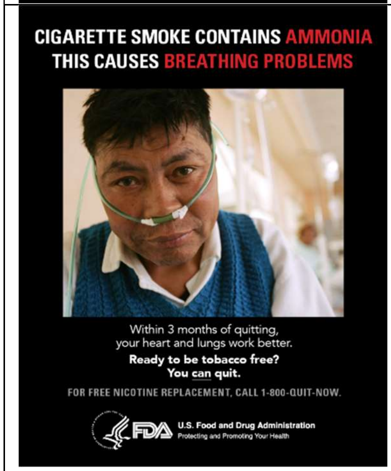

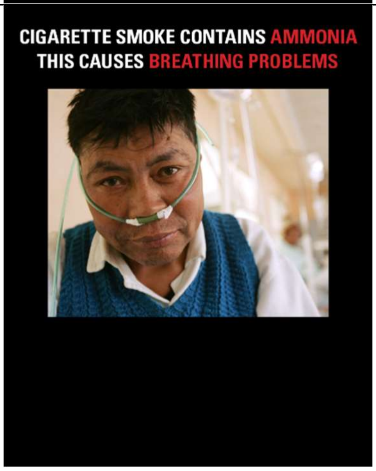

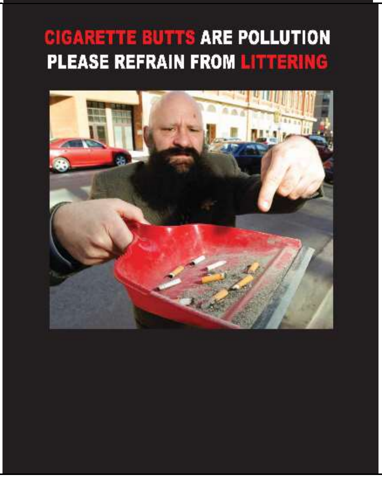

* Images paired with constituent messages are stock images purchased by the researchers, images paired with littering messages are from the following sources (in order https://www.alamedamagazine.com/Oct-2016/From-Ban-to-Butt/, Getty Images stock photo purchased by the researchers, http://www.heraldsun.com.au/leader/inner-south/mordialloc-beach-cleaners-call-for-more-kingston-volunteers-to-beat-the-butt-after-collecting-10000-from-foreshore/news-story/69bd941d20651acbd34a648fe3fe421a, http://www.dailytelegraph.com.au/newslocal/northern-beaches/huge-ciggy-haul-shows-bans-on-manlys-beaches-cant-nip-litter-in-the-butt/news-story/32b8c9c1c53975bf0974091bf6db70f4, https://www.thepeterboroughexaminer.com/news/peterborough-region/2015/04/22/trent-researcher-has-concerns-about-cigarette-butts-in-water-but-it-isn-t-problem-according-to-local-utility.html

© 2021 Goldstein AO et al. *JAMA Network Open.*

eFigure 2. Quit Intentions at Pretest, Day 16, and Day 32

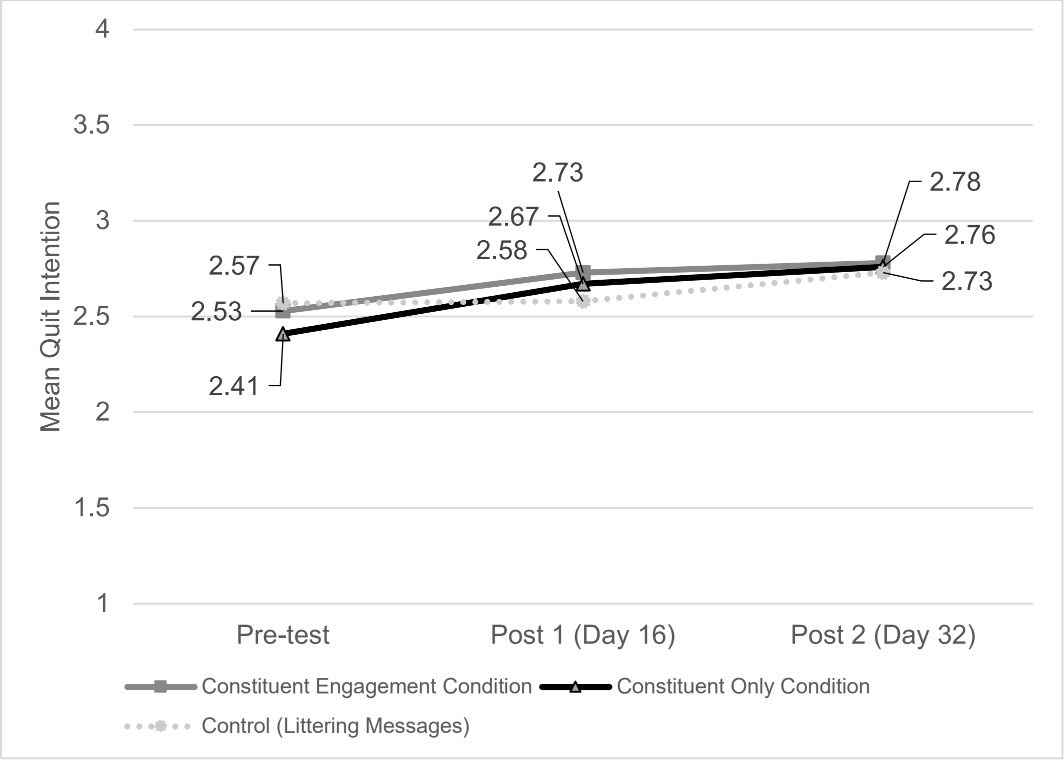

© 2021 Goldstein AO et al. *JAMA Network Open.*

eTable 1. Quit Intentions at Day 16 and Day 32, Without the Dose Variable

	Quit intentions, Day 16			Quit intentions, Day 32		
	**Estimate**	**Standard error**	**p-value**	**Estimate**	**Standard error**	**p-value**
Intercept	0.002	0.05	0.96	**0.16**	**0.06**	**0.006**
Study Condition						
Control condition	REF	REF	REF	REF	REF	REF
Constituent plus engagement condition	**0.19**	**0.07**	**0.004**	0.08	0.08	0.32
Constituent only condition	**0.24**	**0.07**	**0.001**	0.16	0.08	0.05

Boldface denotes statistical significance p < 0.05.

© 2021 Goldstein AO et al. *JAMA Network Open.*

eTable 2. Secondary Behavioral Outcomes at Day 32, Without the Dose Variable

	Cigarettes Smoked			Cigarettes Forgone			Cigarettes Butted Out		
	**Estimate**	**Standard error**	**p-value**	**Estimate**	**Standard error**	**p-value**	**Estimate**	**Standard error**	**p-value**
Intercept	**14.10**	**0.59**	**<.0001**	0.12	0.18	0.49	0.21	0.17	0.23
Study Condition									
Control condition	REF	REF	REF	REF	REF	REF	REF	REF	REF
Constituent plus engagement condition	0.27	0.84	0.75	-0.16	0.26	0.54	-0.14	0.24	0.55
Constituent only condition	-0.91	0.84	0.28	0.09	0.26	0.74	-0.11	0.24	0.64

Boldface denotes statistical significance $p < 0.05$.

© 2021 Goldstein AO et al. *JAMA Network Open.*

eTable 3. Quit Attempts at Day 32, Without the Dose Variable

	aOR	95% CI	p-value
Study Condition			
Control condition (littering messages)	REF	REF	REF
Constituent plus engagement condition	0.96	0.66, 1.41	0.85
Constituent only condition	1.01	0.69, 1.47	0.97
Boldface denotes statistical significance p < 0.05.			

© 2021 Goldstein AO et al. *JAMA Network Open.*

eTable 4. Summary Data on Quit Intentions, Secondary Behavioral Outcomes, and Quit Attempts at Pretest, Day 16, and Day 32

	Constituent plus engagement condition Mean (SD)	Constituent only condition Mean (SD)	Control condition Mean (SD)
Quit intentions			
Baseline	2.5 (0.9)	2.4 (0.9)	2.6 (0.9)
Day 16	2.7 (1.0)	2.7 (0.9)	2.6 (1.0)
Day 32	2.8 (1.0)	2.8 (1.0)	2.7 (1.0)
Number of cigarettes smoked			
Baseline	16.7 (11.0)	14 (10.3)	13.7 (10.9)
Day 16	15.4 (10.2)	12.6 (10.2)	12.6 (10.0)
Day 32	15.9 (9.1)	12.9 (8.4)	13.2 (10.1)
Number of cigarettes forgone			
Baseline	1.4 (3.3)	1.5 (2.4)	1.7 (2.4)
Day 16	1.1 (1.7)	1.5 (1.8)	1.6 (2.1)
Day 32	1.4 (2.2)	1.6 (2.3)	1.7 (2.2)
Number of cigarettes butted out			
Baseline	2.0 (3.5)	1.7 (2.5)	1.7 (2.5)
Day 16	1.9 (2.6)	1.7 (3.5)	1.9 (2.9)
Day 32	2.1 (3.3)	2.3 (3.9)	2.3 (3.2)
Quit attempts			
Baseline	4.3 (24.7)	2.7 (11.4)	2.8 (9.0)
Day 16	0.7 (1.7)	0.7 (1.8)	0.7 (1.9)
Day 32	1.3 (2.6)	1.6 (3.7)	1.6 (3.5)

© 2021 Goldstein AO et al. *JAMA Network Open.*

eTable 5. Quit Attempts at Day 32

	aOR	95% CI	p-value
Number of messages viewed (dose)	**0.95**	**0.90, 0.99**	**0.02**
Study Condition			
Control condition	REF	REF	REF
Constituent plus engagement condition	0.98	0.67, 1.43	0.92
Constituent only condition	1.03	0.71, 1.50	0.88
Boldface denotes statistical significance $p < 0.05$.			

© 2021 Goldstein AO et al. *JAMA Network Open.*

References:

1. National Research Council (US) Panel on Handling Missing Data in Clinical Trials. *The Prevention and Treatment of Missing Data in Clinical Trials*. Washington (DC): National Academies Press (US); 2010

2. Bell ML, Fiero M, Horton NJ, Hsu CH. Handling missing data in RCTs; A review of the top medical journals. *BMC Med Res Methodol*. 2014;14(1):118. doi:10.1186/1471-2288-14-118

3. Jakobsen JC, Gluud C, Wetterslev J, Winkel P. When and how should multiple imputation be used for handling missing data in randomised clinical trials-a practical guide with flowcharts. doi:10.1186/s12874-017-0442-1

4. Noar SM, Hall MG, Francis DB, Ribisl KM, Pepper JK, Brewer NT. Pictorial cigarette pack warnings: a meta-analysis of experimental studies. *Tob Control*. 2016;25(3):341-354. doi:10.1136/tobaccocontrol-2014-051978

5. Rubin, D. B. (1987). Multiple Imputation for Nonresponse in Surveys. New York: John Wiley & Sons. https://onlinelibrary.wiley.com/doi/pdfdirect/10.1002/9780470316696.fmatter Accessed August 20, 2020.

© 2021 Goldstein AO et al. *JAMA Network Open.*
