# Supplementary material for: Effect of Cigarette Constituent Messages With Engagement Text on Intention to Quit Smoking Among Adults Who Smoke Cigarettes: A Randomized Clinical Trial
Source: JAMA Netw Open. 2021 Feb 24;4(2):e210045. doi: 10.1001/jamanetworkopen.2021.0045 (PMC7905497; doi:10.1001/jamanetworkopen.2021.0045)
Supplement: Supplement 3. — Data Sharing Statement [file jamanetwopen-e210045-s003.pdf]

Data Sharing Statement

Goldstein. Effect of Cigarette Constituent Messages With Engagement Text on Intention to Quit Smoking Among Adults Who Smoke Cigarettes. *JAMA Netw Open*. Published February 24, 2021. doi:10.1001/jamanetworkopen.2021.0045

Data
Data available: Yes
Data types: Deidentified participant data
How to access data: https://clinicaltrials.gov/ct2/show/study/NCT03339206
When available: With publication

Supporting Documents
Document types: Statistical/analytic code
How to access documents: jkristen@email.unc.edu
When available: With publication

Additional Information
Who can access the data: anyone requesting the data
Types of analyses: analysis to confirm results
Mechanisms of data availability: with a signed data access agreement
